# CNN-LSTM-AM approach for outdoor wireless optical communication systems

**DOI:** 10.1038/s41598-025-16828-2

**Published:** 2025-09-01

**Authors:** Montaser Abdelsattar, Eman S. Amer, Hamdy A. Ziedan, Wessam M. Salama

**Affiliations:** 1 Electrical Engineering Department, Faculty of Engineering, South Valley University, Qena 83523, Egypt; 2 Faculty of Industrial and Energy Technology, Borg Al Arab Technological University, Alexandria, Egypt; 3 Department of Electrical Engineering, Faculty of Engineering, Assiut University, Assiut, 71516 Egypt; 4 Electrical Engineering Department, Faculty of Engineering, Pharos University, Canal El Mahmoudia Street, Beside Green Plaza Complex 21648, Alexandria, Egypt; 5 Department of Computer Engineering, Faculty of Engineering, Pharos University, Canal El Mahmoudia Street, Beside Green Plaza Complex 21648, Alexandria, Egypt

**Keywords:** Deep learning, Bit error rate (BER), Deep denoising autoencoder (DDAE), Convolutional neural network (CNNs), Generative adversarial network (GAN), Gated recurrent unit (GRU), Engineering, Electrical and electronic engineering

## Abstract

This paper introduces the enhancement of Visible Light Communications (VLC) for V2V using artificial intelligence models. Different V2V scenarios are simulated. The first scenario considers a specific longitudinal separation and a variable lateral shift between vehicles. The second scenario assumes random longitudinal separation and a specific lateral shift between vehicles. Significant obstacles that impair performance and dependability in V2V communication systems include bit errors, high power consumption, and interference. By combining Convolutional Neural Networks (CNNs), Generative Adversarial Network (GAN), Gated Recurrent Unit (GRU), and Deep Denoising Autoencoder (DDAE), this paper suggests a deep learning-based system to address these issues. The framework comprises four modules, a power reduction module that uses a GAN to generate low-power signals while maintaining signal quality; a performance enhancement module that uses GRU, a Bit Error Rate (BER) reduction module that uses a DDAE to denoise the received signal and minimize errors; and an interference cancellation module that uses a CNN-based U-Net to separate the desired signal from interference. It is shown that the suggested model significantly improves throughput, power efficiency, BER reduction, and interference cancellation. In dynamic and noisy contexts, our study offers a reliable and scalable way to improve the performance and dependability of V2V communication systems. The CNN-U-Net-GAN-GRU-DDAE model outperforms other models, including CNN-U-Net, CNN-U-Net-GAN, and CNN-U-Net-GAN-GRU, achieving the best results by an average percentage 13.6%, 14.4% and 4.2% respectively. By comparing this work with previous works, we deduce that the improving average percentage for our work by 31.7%.

## Introduction

The revolution of the wireless communication system with six-generation technologies presents a several applications as visible light communications (VLC), Multiple-Input Multiple-Output (MIMO), millimeter wave and other applications to enhance the performance of communication systems^1^. The VLC system outperforms Radio Frequency (RF) for its advantages as large spectrum and high data rate with high level of security, so VLC is considered as a promising technology^2^. Also, applied Light-Emitting Diodes (LEDs) offer high efficiency in both energy and spectrum and low cost^3^. LEDs utilize intensity modulation with a high data rate that enhances the capabilities of the wireless communication^4^. Thus, VLC is considered a green communication technology^5^.

To improve the performance of VLC, both of VLC and RF as a hybrid communication system are utilized^6^. Furthermore, VLC can be used in restricted areas where RF cannot be used as airplanes and radiation department in hospitals where VLC uses a different range of frequencies so don’t cause any interference as done with RF^7^.

Recent research on VLC systems has been conducted to enhance the system performance with different modulations^8^, and different coding^9^, and utilizing MIMO and MISO systems^10^, transceiver design^11^, pre-equalization and post-equalization^12,13^, and channel capacity for dimmable VLC^14^. Transport intelligent systems, IoT, street level access networks are important applications in 5G wireless communications systems utilizing VLC systems, where transport intelligent systems were presented by applying VLC and positioning systems^15^. Automotive applications were proposed for modeling the channels which suffers from reflections when using VLC in transport intelligent systems^16^. Authors in Ref.^17^ proposed environmental-adaptive receiver for performance enhancement of automotive applications.

The previous literature review didn’t concern the random mobility of the vehicle, random lateral shift of the vehicle, random longitudinal separation of the vehicle which this random mobility causes random path loss modeling of the joint impact of path loss and atmospheric turbulence.

In Ref.^18^, the authors supposed a dynamic V2V scenario where vehicles differ in their positions related to several parameters such as the layout of road, speed, and other nearby mobile vehicles. This dynamical surround V2V impacts the performance of the system due to short range of transmission the lighting signal. Thus, this research assumes some properties of V2V system as random mobility, random path loss, random lateral shift and random longitudinal separation where these properties can improve the accuracy of the system model.

**The main contributions in this work can be summarized in the following points**:

- Related to the presented scenarios in Ref.^18^, the datasets are collected to create the deep learning models of the V2V- VLC system.
- Combining CNNs, GAN, GRU and DDAE models are proposed in this paper.
- The GAN is used to generate low-power signals and improve the quality of the signals.
- Moreover, the GRU enhances the system’s performance.
- Furthermore, the DDAE is performed to reduce the BER by denoising the received signal, minimizing the errors.
- The CNN-based U-Net is applied to cancel the interference of the modules.
- In general, the models are used to improve the throughput, power efficiency, BER reduction, and interference cancellation.
- Dynamic systems are introduced to provide reliable and scalable methods to enhance the performance.

The remainder of the paper is structured as follows. The system model is discussed in Sect. “System model”. The methodologies used in the paper are listed in Sect. “Methodology”. Results and analysis based on simulation and assessment parameters are displayed and explained in Sect. "Results and discussion". The findings are concluded in Sect. “Conclusion”, which also lists suggestions for future work.

## System model

This work proposes V2V with VLC modeling in two parallel roads. The effect of Atmospheric Turbulence (AT) and different parameters with conditions of random lateral shift and the longitudinal separation as discussed in Ref.^18^ and are shown in Table 1. Two scenarios are assumed first, scenario 1 with the random lateral shift of vehicles and deterministic longitudinal separation between two vehicles; and second, scenario 2 with random longitudinal separation between two vehicles and deterministic lateral shift of vehicles.

**Table 1:** Parameters of V2V – VLC system.

Notation used in this paper	
W_L_	Width of single lane
W_v_	Width of vehicle
D	Longitudinal separation between the two vehicles
d_h_	Lateral shift of the vehicle
φ_1_, φ_2_	Angles of irradiance w.r.t $\:{T}_{x1}$ and $\:{T}_{x2}$
$\:\varvec{\theta\:}$_1_, $\:\varvec{\theta\:}$_2_	Angles of incidence w.r.t $\:{T}_{x1}$ and $\:{T}_{x2}$
L_i_	Propagation distance from the i^th^ transmitter ($\:{T}_{x}$)
Ψ	Field-of-view (FOV)angle of the receiver ($\:{R}_{x}$)
D_R_	Aperture diameter of the $\:{R}_{x}$

**Fig. 1:**
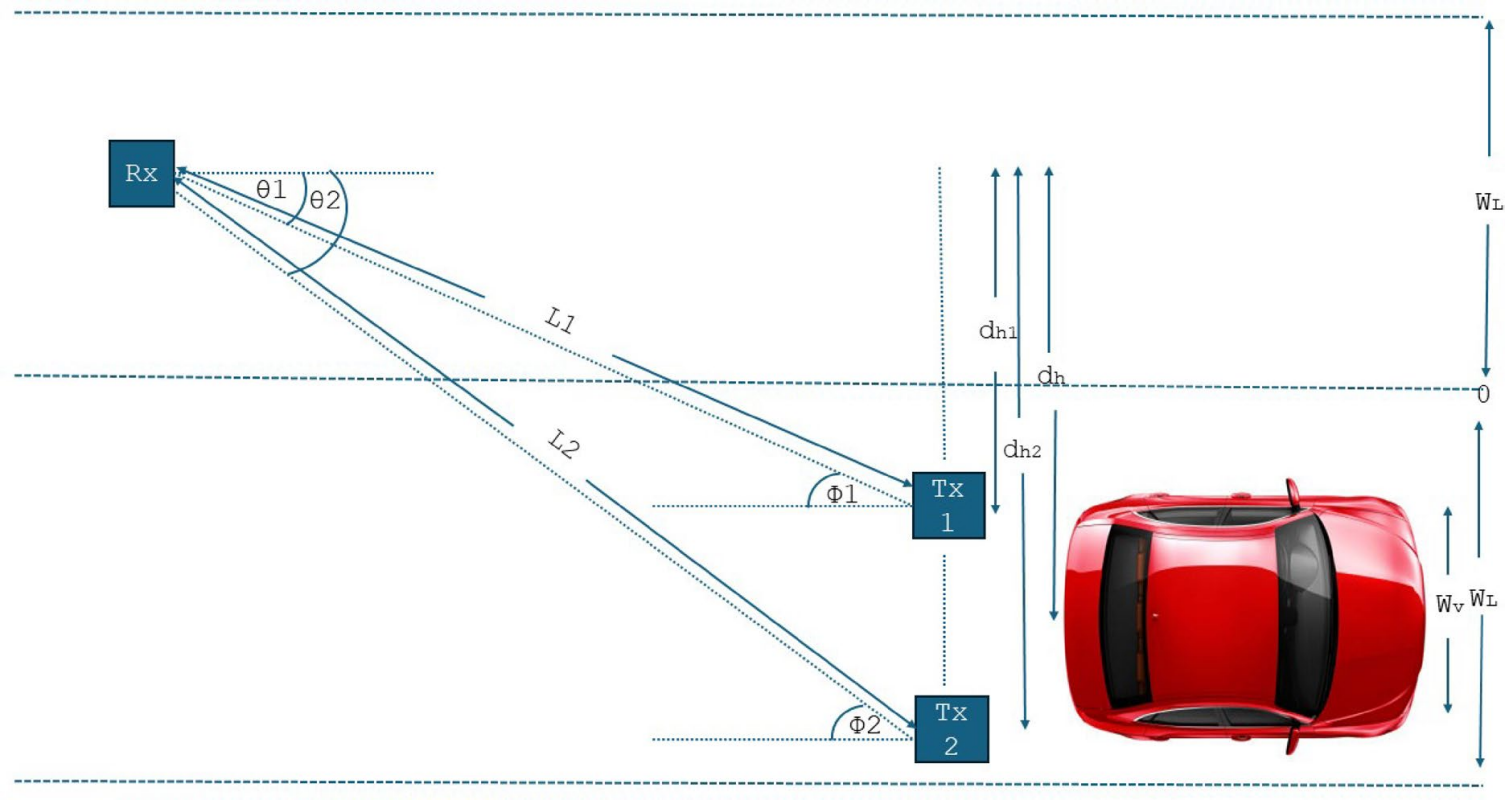
The Suggested V2V-VLC Communication Framework System Model.

In this side, the V2V-VLC and channel models are discussed. Two parallel highways or two-lane roads with two vehicles encountered in both lanes are expected, as illustrated in Fig. 1. One car in one lane uses the transmitter in its backlight, and another car in a different lane uses the photodetector at its bumper as the receiver.

According to the geometry in Fig. 1, φ_1=_ $\:\theta\:$_1_ and φ_2=_ $\:\theta\:$_2_.

The lateral shift related to i^th^ transmitter can be expressed as follows:

1 $$\:{d}_{h}=\:{d}_{h}\:\pm\:\frac{{w}_{v}}{2},\:i=\text{1,2},\dots\:\dots\:\dots\:.$$

The propagation distance Li for i^th^ is defined as follows:

2 $$\:{L}_{i}=\sqrt{{d}^{2}+{d}_{h}^{2}}$$

The incident angle depends on d_h_ as the following

3 $$\:{\theta\:}_{i}=\:{cos}^{-1}\left(\frac{d}{{L}_{i}}\right)$$

$\:{d}_{h}$is random value can be varied from 0 to (2W_L_-W_v_).

Based on the analysis in Ref.^18^, it is found

4 $$\:\text{cos}\left({\theta\:}_{i}\right)\ge\:\text{cos}\left({\Psi\:}\right)$$

, and,

5 $$\:{L}_{i}\le\:\frac{d}{\text{cos}\left({\Psi\:}\right)}$$

Also, it is deduced that,

6 $$\:{d}_{hi}^{2}\le\:{d}^{2}\left(\frac{1-{cos}^{2}\left({\Psi\:}\right)}{{cos}^{2}\left({\Psi\:}\right)}\right)$$

Therefore, $$\:{d}_{hi}\le\:d\:\text{t}\text{a}\text{n}\left({\Psi\:}\right)$$ (7)

8 $$\:{d}_{hi}=\:{d}_{h}\pm\:\:\frac{{W}_{v}}{2}$$

By substituting in the equations, the following equations can be obtained as follows:

9 $$\:\left({d}_{h}\pm\:\frac{{W}_{v}}{2}\right)\le\:d\:tan\left({\Psi\:}\right)$$

For d_h1_:

10 $$\:{d}_{h}-\:\frac{{W}_{v}}{2}\le\:d\text{tan}\left({\Psi\:}\right)$$

11 $$\:{d}_{h}\le\:d\text{tan}\left({\Psi\:}\right)+\:\frac{{W}_{v}}{2}$$

If the previous equation is satisfied then L_1_ is detected by the receiver.

For d_h2_:

12 $$\:{d}_{h}+\:\frac{{W}_{v}}{2}\le\:d\text{tan}\left({\Psi\:}\right)$$

13 $$\:{d}_{h}\le\:d\text{tan}\left({\Psi\:}\right)-\:\frac{{W}_{v}}{2}$$

If the previous equation is satisfied then L_2_ is detected by the receiver.

Thus, the general condition that

14 $$\:{d}_{h}\le\:d\text{tan}\left({\Psi\:}\right)\pm\:\:\frac{{W}_{v}}{2}$$

The channel modeling of the V2V – VLC can be characterized as the following: the received signal by the vehicle in lane 2 can be expressed as follows:

15 $$\:y=\:\eta\:\sum\:_{i=1}^{2}{h}_{i}x+e,$$

Where y is received signal, $\:\eta\:$ is responsivity of the photodetector, x is positive value for VLC signal between transmitter and receiver, e is considered additive white Gaussian noise.

Note that $\:{h}_{i}$ is real channel coefficient between transmitter and receiver as follows:

16 $$\:{h}_{i}=\:{h}_{ai}{\left({{h}_{i}}^{PL}\right)}^{avg}$$

Where $\:{h}_{ai}$ express AT that include the effects of the surrounding conditions of the environment, $\:{\left({{h}_{i}}^{PL}\right)}^{avg}$ is the average pathloss.

## Methodology

**Fig. 2:**
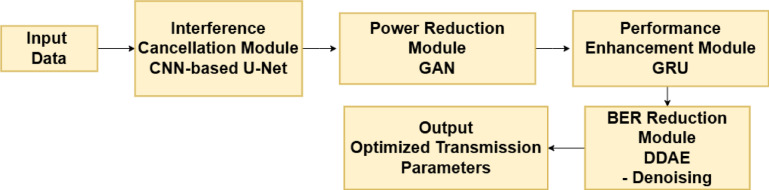
Block Diagram of the Suggested Framework Based on Deep Learning.

To mitigate interference from other vehicles or environmental sources, lower transmission power while maintaining dependable communication, improve overall system performance, including throughput and reliability, and eventually lower the BER by enhancing signal quality and minimizing data transmission errors, the suggested model combines Convolutional Neural Networks (CNNs), Recurrent Neural Networks (RNNs), and Generative Adversarial Networks (GANs). In our proposed framework, the workflow consists of the input raw received signal, which includes the desired signal, noise, and interference. Channel State Information (CSI) is another name for data pertaining to the communication channel. A CNN model was used to identify interference patterns and extract spatial features from the received information. Additionally, a U-Net model is used to distinguish between interference and the intended signal. Furthermore, the GAN is used to preserve the information content of the sent signal while producing a low-power version. The discriminator ensures that the signal quality is preserved, whereas the generator is used to teach the GAN to learn a signal that uses less power. The GRU is used to maximize the signal transmission and model temporal dependencies in the communication channel. Finally, to eliminate noise and clean up the received signal, a Deep DDAE is used to reduce the BER by training the DDAE to reconstruct the original signal from the noisy input. This is illustrated in Fig. 2.

### Dataset description

The CNNs, GAN, GRU and DDAE models are trained using the datasets, which were extracted as shown in Ref.^18^. These datasets provide crucial statistics for two scenarios as shown in system model. A comprehensive dataset comprising 155 vectors of transmitted signals propagated through an outdoor channel which is used to train and evaluate the suggested models. The dataset is divided into 70% training and 30% testing and validation 15% validation and 15% testing) in order to train the deep learning models. Datasets with pathloss, BER and SNR values are used to optimize the models during the training stage.

### Interference cancellation module (The CNN module based on the U-Net Model)

#### Purpose and overview

An essential part of the suggested deep learning framework for V2V communication systems is the interference cancellation module. Its goal is to isolate the intended signal from interference in the received signal, which is crucial for performance enhancement and dependability of communication. A detailed explanation of the module is explained in this paper emphasizing the application of CNN and the U-Net architecture.

#### Feature extraction based on the CNN

The module first step is to extract spatial information from the incoming signal using a CNN. The patterns in the data is automatically learnt and recognized based on CNN. The received signal $\:y(t$), which is commonly represented as a time-frequency representation, serves as the CNN input. From the incoming signal, the CNN learns to extract interference patterns and spatial characteristics. Furthermore, the target signal is separated from interference using the U-Net architecture following the extraction of spatial characteristics. A particular kind of CNN called U-Net is created especially for picture segmentation, though it can also be modified for signal processing applications as expressed in the following equation:

17 $$\:{G}_{m}\left(n\right)=\sigma\:(\sum\:_{k}{w}_{m,k}.z\left(n-k\right)+{b}_{m}$$

where $\:G$ is the feature map output from the convolutional layer, $\:{w}_{m,k}$ is the weights of the $\:{m}^{th}\:$filters at position $\:k$, $\:{b}_{m}$ and $\:\sigma\:$ represent the bias term of the $\:{m}^{th}\:$filters and the activation function, respectively.

To reduce the dimensionality of the feature, map the Max- Pooling is used as follows:

18 $$\:{P}_{m}\left(n\right)=\text{max}{G}_{m}\left(n-k\right)$$

where $\:K=3$ which is the pooling window which is implemented in this paper.

**Encoder (Down-Sampling Path)**.

The intended signal $\:s\left(t\right)$ is separated from noise $\:n\left(t\right)\:$and interference $\:i\left(t\right)$ using the U-Net architecture. High-level characteristics are extracted from the input signal by the encoder. The encoder layers are made up of convolution, pooling and the bottle neck as follows:

19 $$\:{G}_{l}\left(n\right)=\sigma\:({w}_{l}*{G}_{l-1}\left(n\right)+{b}_{l})$$

20 $$\:{P}_{l}\left(n\right)=pool\:\left({G}_{l}\left(n\right)\right)$$

21 $$\:B\left(n\right)=\sigma\:({w}_{b}*{P}_{L}\left(n\right)+{b}_{b})$$

where $\:B\left(n\right)$ is the most features that captured by the convolutional layers, $\:L$ is the number encoder layers with initial weight and bias value $\:{w}_{1}=2$, $\:{b}_{1}=1$, $\:{w}_{b}=1.5$ and $\:{b}_{b}=2$.

**Decode (Up-Sampling Path)**.

The output signal is reconstructed by the decoder using the features that are extracted. Transposed convolution (deconvolution), skip connections that concatenate feature mappings from the encoder to the decoder, and the output layer make up each layer of the decoder. These are expressed as follows:

22 $$\:{D}_{l}\left(n\right)=\sigma\:({w}_{l}^{M}*{D}_{l+1}\left(n\right)+{b}_{l})$$

23 $$\:{D}_{l}\left(n\right)=concat({D}_{l}\left(n\right),{G}_{l}\left(n\right)\:)$$

In this paper, $\:{w}_{2}^{M}=1.2$ and $\:{b}_{2}=1$ while $\:{w}_{1}^{M}=1.5$ and $\:{b}_{1}=2$.

Finally, the estimated desired signal is:

24 $$\:\widehat{s}\left(n\right)=\sigma\:({w}_{0}*{D}_{1}\left(n\right)+{b}_{0})$$

**Loss Functions and Evaluation Metrics**.

Based on the following loss functions, the U-Net is trained to reduce the discrepancy between the estimated signal $\:\widehat{s}\left(n\right)$ and the actual desired signal $\:s\left(n\right)$:

25 $$\:{\mathcal{L}}_{MSE}=\frac{1}{C}\:\sum\:_{n=1}^{C}{(s\left(n\right)-\widehat{s}\left(n\right))}^{2}$$

26 $$\:{\mathcal{L}}_{SNR}=-10\:{\text{log}}_{10}\left(\frac{\sum\:_{n=1}^{C}{s\left(n\right)}^{2}}{\sum\:_{n=1}^{C}{(s\left(n\right)-\widehat{s}\left(n\right))}^{2}}\right)$$

where MSE is the mean square error and the SNR is the signal to noise ratio.

Lastly, the Interference Cancellation Ratio (ICR) and the Signal to Interference Ratio (SIR) metrics are used to assess the model on the test dataset:

27 $$\:ICR=10{\text{log}}_{10}(\frac{\sum\:_{n=1}^{C}{i\left(n\right)}^{2}}{\sum\:_{n=1}^{C}{\widehat{i}\left(n\right)}^{2}})$$

28 $$\:SIR=10{\text{log}}_{10}(\frac{\sum\:_{n=1}^{C}{s\left(n\right)}^{2}}{\sum\:_{n=1}^{C}{\widehat{i}\left(n\right)}^{2}})$$

#### Network configuration and hyperparameters

**Fig. 3:**
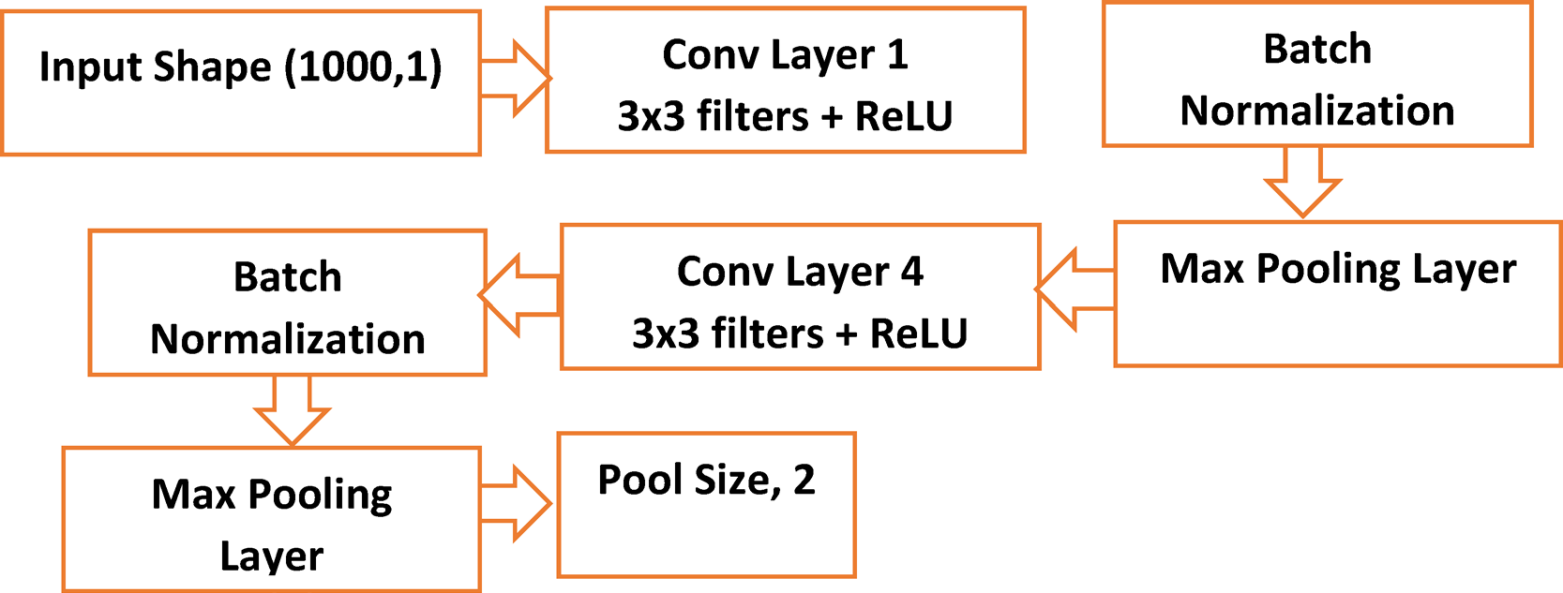
Flowchart of the CNN Framework.

The CNN module input shape is (1000, 1), as shown in Table 2; Fig. 3. A deeper feature extraction method is indicated by the four convolutional layers with progressively larger filter sizes [32,64,128,256]. Hierarchical spatial properties can be effectively captured in this way. A good kernel size of three strikes a balance between computational efficiency and local feature extraction. Vanishing gradient problems are lessened by the Swish activation, which aids in the model’s learning of intricate patterns. Furthermore, an excellent down sampling technique that maintains significant features is Max-Pooling with a pool size of two. A crucial component of the U-Net architecture, symmetry is maintained by the encoder and decoder in the U-Net module, each of which has four layers. Progressive feature extraction and subsequent reconstruction are indicated by the filter sizes in the encoder [64,128, 256, 512] and decoder [256,128, 64, 32]. Moreover, the ELU activation is suitable, smoother gradients may be advantageous.

**Table 2:** The CNN- U-Net module numerical parameters.

Parameter	Value
CNN Module	
Input Shape	(1000, 1)
Number of Convolutional Layers	4
Filters	[32, 64, 128, 256]
Kernel Size	3
Activation Function	Swish
Pooling	Max Pooling
Pool Size	2
U-Net Module	
Encoder Layers ($\:\varvec{L}$)	4
Decoder Layers	4
Filters in Encoder	[64, 128, 256, 512]
Filters in Decoder	[256, 128, 64, 32]
Kernel Size	3
Activation Function	ReLU
Output Filters	1
Output Activation	Linear

#### Training configuration

**Fig. 4:**
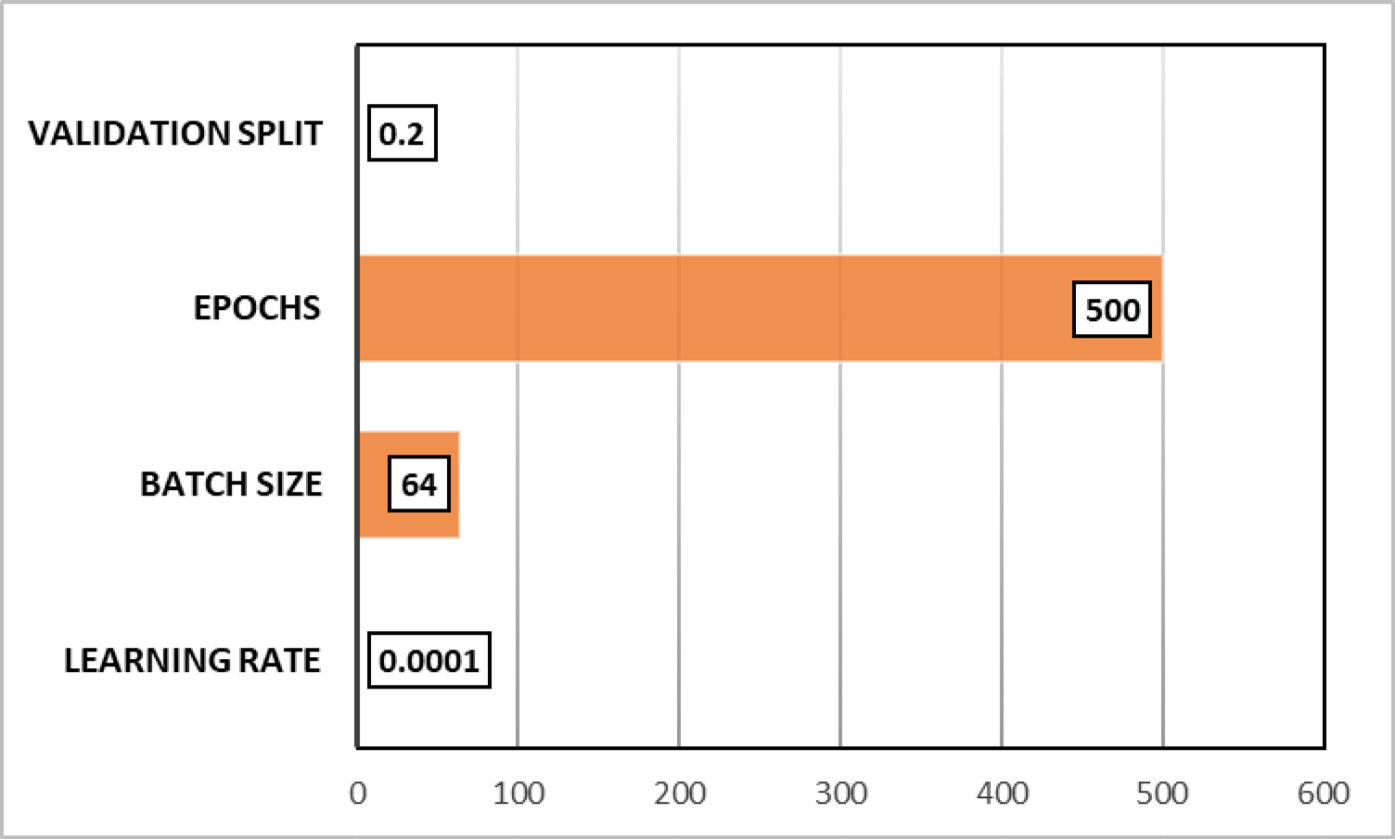
Training Hyperparameters and Optimization Settings.

The selected hyperparameters define the model training behavior and optimization strategy, as illustrated in Fig. 4. Adaptive Moment Estimation (Adam), which combines momentum and adaptive learning rates to generate reliable updates and accelerate convergence, is an excellent choice. A small learning rate of 0.0001, which ensures continuous convergence, may lead to longer training sessions. A batch size of 64 is used to balance processing efficiency with consistent gradient changes. A 20% validation split is utilized in order to generalize the model’s capabilities.

### The integration between the CNN module based on the U-Net model and the GAN module

#### Motivation for integration

In V2V communication systems, the interference cancellation module is improved by combining a GAN with the CNN-U-Net model.

#### Power reduction module (GAN Architecture)

**Generator (G)**

A generative component added by the GAN can replicate genuine interference patterns and increase the model resilience. The generator (G), one of the two networks that make up the GAN, creates artificial data (interference patterns) to supplement the training dataset and increase the model’s capacity for generalization.

**Discriminator (D)**

**Fig. 5:**
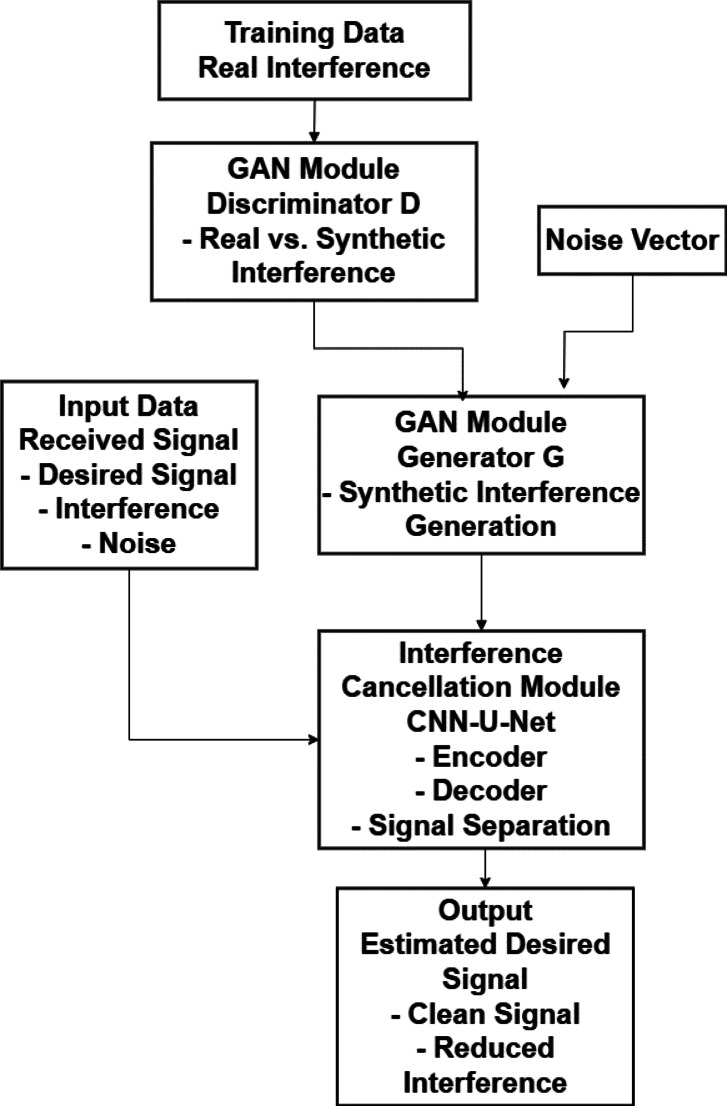
Workflow of the Integrated CNN-U-Net-GAN Module.

The discriminator (D), which distinguishes between real data (like actual interference) and fake data generated by G. The realistic interference patterns generated in the proposed framework are used to train the introduced model. The G generates a low-power version of the transmitted signal while maintaining signal quality. As shown in Fig. 5, the D ensures the realistic and high-quality generated signal.

### The CNN-U-Net-GAN-GRU module

#### Overview and purpose

The performance enhancement module of the suggested CNN-U-Net-GAN framework for V2V communication systems depends heavily on the GRU.

#### Performance enhancement module (GRU Role)

The GRU is used to dynamically optimize transmission settings and represent temporal dependencies in the communication channel. The GRU is used in the presented system to record temporal dependencies in the communication channel, including noise changes and interference patterns. To enhance system performance, adjust the transmission parameters (power level) in response to real-time feedback. In this paper, the low-power signal from the GAN and the cleaned signal from the CNN-U-Net are sent to the GRU. After that, it optimizes the transmission settings by processing the sequential data. The system receives the optimum transmission parameters from the GRU and uses them to adjust in real time. The CNN-U-Net generates the estimated desired signal $\:\widehat{s}\left(n\right)$ by processing the received signal $\:y\left(n\right)$. The low-power signal $\:{x}_{low}\left(n\right)$ is produced by the GAN. The input vector $\:x\left(n\right)$ for the GRU is created by combining the outputs $\:\widehat{s}\left(n\right)$ and $\:{x}_{low}\left(n\right)$). The GRU optimizes transmission parameters $\:{a}_{n}$ by processing $\:x\left(n\right)$ based on the following:

29 $$\:\widehat{s}\left(n\right)=CNN-U-Net\left(y\left(n\right)\right)$$

30 $$\:{x}_{low}\left(n\right)=G\left(Z\right)$$

31 $$\:x\left(n\right)=[\widehat{s}\left(n\right),\:{x}_{low}\left(n\right)]$$

Finally, the GRU output is as follows:

32 $$\:{a}_{n}={w}_{0}.{h}_{t}+{b}_{0}$$

where the initial weight and bias are set to be $\:{w}_{0}=1.2\:and\:{b}_{0}=0.5$.

#### Network parameters

**Fig. 6:**
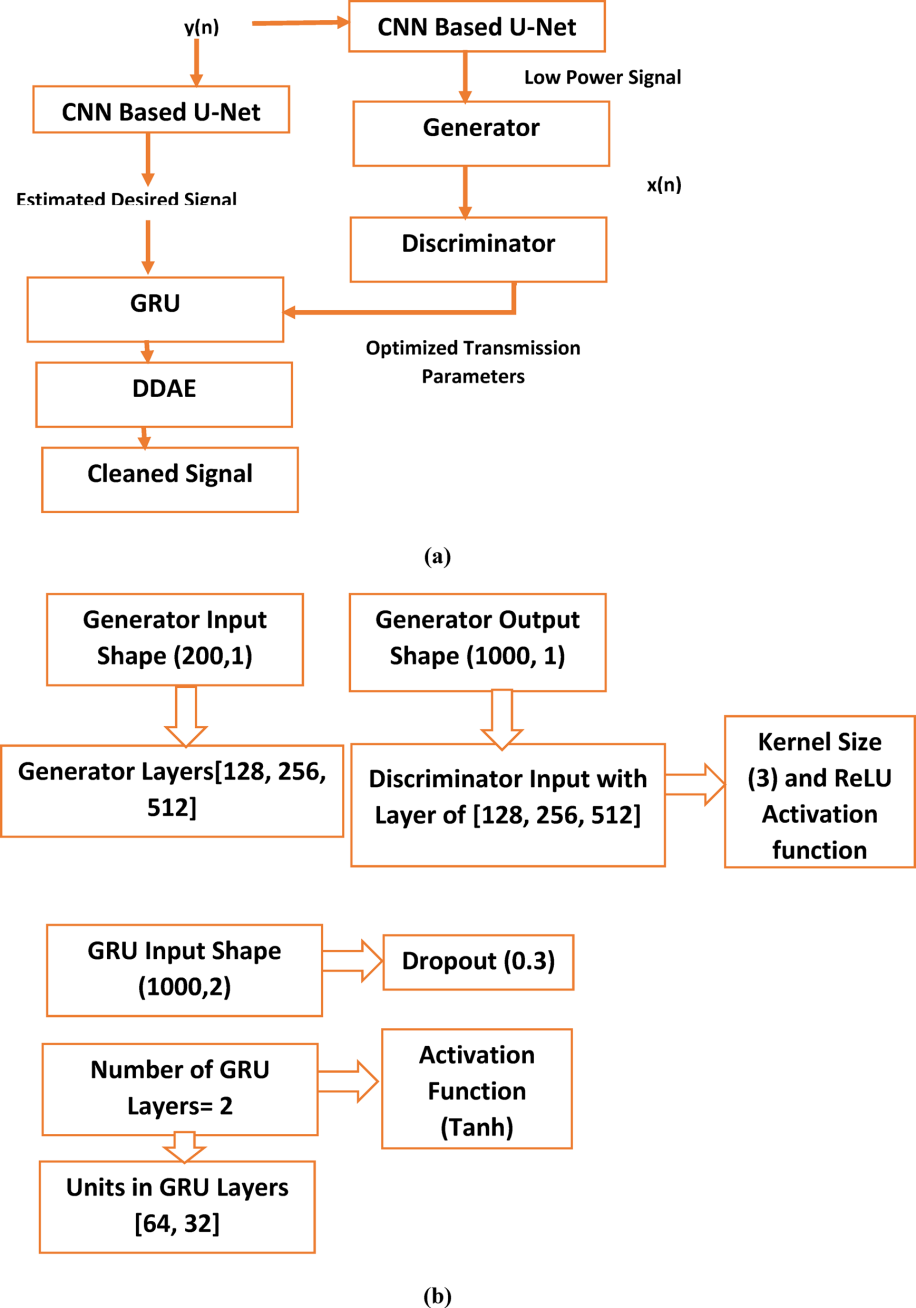
Flowchart of the GAN, GRU Framework.

As seen in Table 3 and Fig. 6, the generator takes in a 200-dimensional latent space vector, which gives enough randomness to develop a diverse distribution. The output shape (1000, 1) indicates that the generator creates a 1000-sample-long 1D signal, which is well-aligned for our dataset. Gradual feature extraction and transformation is made possible by the generator layer progression [128, 256, 512], which guarantees that the generated output captures improve system performance. The discriminator uses hierarchical down-sampling [64, 128, 256], which efficiently compresses input signals while learning to differentiate between real and fake samples. There are two features in each of the 1000 time increments that make up the input. The model capacity to identify both short-term and long-term dependencies in sequential data is improved by the use of two GRU layers. Low-level patterns are captured by the first GRU layer, and higher-level dependencies are the focus of the second layer. A 32-unit GRU layer that reduces dimensionality while preserving key characteristics comes after the 64-unit initial layer that extracts intricate temporal relationships. By keeping activations centered around zero, the Tanh activation function aids in gradient flow and avoids saturation problems. By introducing dropout of 0.3 in between GRU layers to enhance generalization and prevent the overfitting.

**Table 3:** The GAN simulation parameters.

Parameter	Value
GAN	
Generator Input Shape	(200, 1)
Generator Output Shape	(1000, 1)
Generator Layers	[128, 256, 512]
Discriminator Input Shape	(1000, 1)
Discriminator Layers	[64, 128, 256]
Kernel Size	3
Activation Function	ReLU
GRU	
Input Shape	(1000, 2)
Number of GRU Layers	2
Units in GRU Layers	[64, 32]
Output Shape	(2,)
Activation Function	Tanh
Dropout	0.3

### The CNN-U-Net-GAN-GRU-DDAE module

#### Overview and objective

By including a DDAE as a BER reduction module into the CNN-U-Net-GAN-GRU framework, the system capacity to reduce errors and denoise the incoming signal is improved. In order to improve signal quality, rebuild the clean signal, and lower the BER, the DDAE is utilized to eliminate noise and interference from the received signal.

#### BER reduction module (DDAE functional Role)

The DDAE learns a mapping in order to recover $\:s\left(n\right)$from $\:y\left(n\right)$:

33 $$\:{f}_{\theta\:}\left(y\right)\approx\:s$$

where $\:{f}_{\theta\:}$ is the trained DDAE model.

In order to extract the most significant features and eliminate noise, the encoder compresses the received noisy signal $\:y$ into a latent representation $\:h$ as follows:

34 $$\:h={f}_{e}\left(y\right)=\sigma\:({W}_{e}y+{b}_{e})$$

where $\:{W}_{e}$ and $\:{b}_{e}\:$are the encoders weight matrix and bias vector which are initialize our model, $\:\sigma\:$ is the activation function and $\:h\:$represents the input latent representation.

#### Decoder architecture

While the decoder reconstructs the denoised signal $\:\widehat{s}\left(n\right)$ from the latent representation $\:h$ as follows:

35 $$\:\widehat{s}\left(n\right)={f}_{d}\left(h\right)=\sigma\:({W}_{d}h+{b}_{d})$$

where $\:{W}_{d}$ and $\:{b}_{d}$ are the decoder weights and biases.

#### DDAE architecture and parameters

**Fig. 7:**
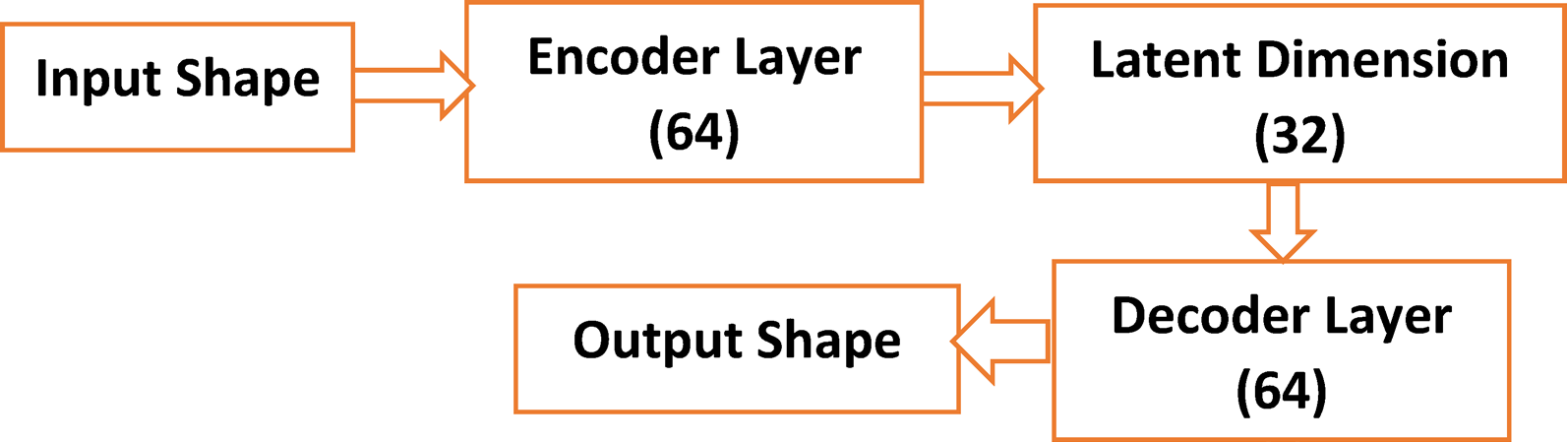
Flowchart of the DDAE Framework.

As seen in Table 4; Fig. 7, the encoder accepts a one-dimensional input sequence of 1000 samples, as shown by the input shape (1000, 1). The input data is compressed into a lower-dimensional latent form using encoder layers [64, 32]. A progressive decrease in the dimensionality of the input data is suggested by the neurons’ reduction from 64 to 32. The encoder converts the 1000-sample input into a 32-dimensional vector when the latent dimension is 32. The latent dimension 32 values are simulated in the introduced framework for the 1000 input shape values, resulting in a notable reduction in dimensionality. From the latent representation, the original input data is reconstructed using decoder layers of [32, 64].

**Table 4:** The numerical parameters for the DDAE.

Parameter	Value
Input Shape	(1000, 1)
Encoder Layers	[64, 32]
Latent Dimension	32
Decoder Layers	[32, 64]
Output Shape	(1000, 1)
Activation Function	ReLU
Loss Function	MSE
$\:{\:\varvec{W}}_{\varvec{d}}$	$\:[0.4,\:0.6]$
$\:{\varvec{b}}_{\varvec{d}}$	0.1.
$\:{\varvec{W}}_{\varvec{e}}$	$\:[0.5,\:0.3]$
$\:{\varvec{b}}_{\varvec{e}}$	$\:0.2$

#### Workflow summary and evaluation

**Fig. 8:**
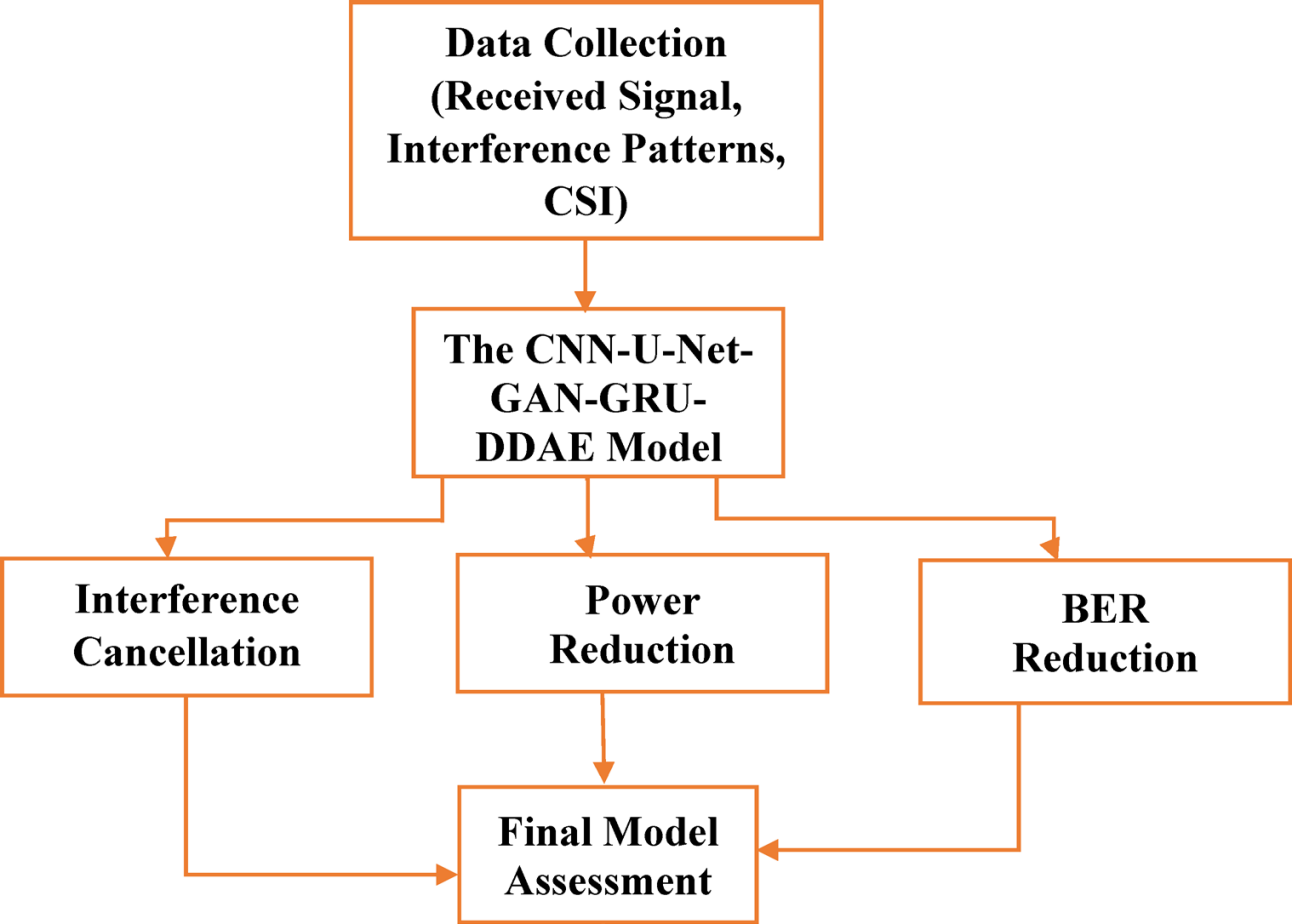
Flowchart of the Complete CNN-U-Net-GAN-GRU-DDAE Framework.

Figure 8 effectively outlines a structured workflow for evaluating a deep learning-based interference cancellation and BER reduction system, including data collection, model training, evaluation, and final assessment. BER, Power Reduction Ratio (PRR), and ICR are among the important performance measures that are used.

## Results and discussion

In this section, the numerical results and discussion are illustrated with figures as derived in^18^ by utilizing the four proposed models. Further, at a distance of 40 m, the path loss for both moderate and dense of the fog weather scenarios can be 2 and 3 dB, respectively. Furthermore, the combined impact of path loss and atmospheric turbulence affects the V2V-VLC performance significantly. Furthermore, Python program is implemented to simulate the results and compare the enhancement of our results with that shown in previous work^18^ to determine the improvement percentages and to know which of the proposed models has the best results over others.

As discussed in Ref.^18^, the same parameters are used for V2V – VLC model as shown in Table 5.

**Table 5:** Simulation parameters for the proposed V2V- VLC model^13,18^.

Parameter	Value
W_L_	5 m
W_v_	2.5 m
P_t_	10 W
n_d_	10
P	0.5
D	20 m (ABER for scenario1)
d_h_	2 m (ABER for scenario2)
D_R_	5 cm
Ψ	180^o^

As seen in Table 6, the improvement in ICR and SIR is computed for every model upgrade in order to measure the performance. The CNN-U-Net-GAN obtains a 16.3% rise in SIR and a 28.5% improvement in ICR when compared to the baseline model. Additionally, the CNN-U-Net-GAN-GRU improves SIR by 4.8% and ICR by 15.9%. The CNN-U-Net-GAN-GRU-DDAE, which increases SIR by 22.6% and ICR by 20.6%, finally achieves the greatest improvement. Overall, the ICR and SIR are improved by 79.6% and 49.5%, respectively, from the baseline CNN-U-Net to the final CNN-U-Net-GAN-GRU-DDAE model, indicating the proposed model’s notable efficacy in lowering interference and improving signal quality.

**Table 6:** The proposed model performance.

Model	ICR (dB)	SIR (dB)
CNN-U-Net	13.7	19.6
CNN-U-Net-GAN	17.6	22.8
CNN-U-Net-GAN-GRU	20.4	23.9
CNN-U-Net-GAN-GRU-DDAE	24.6	29.3

1.CNN-U -Net model.

**Fig. 9:**
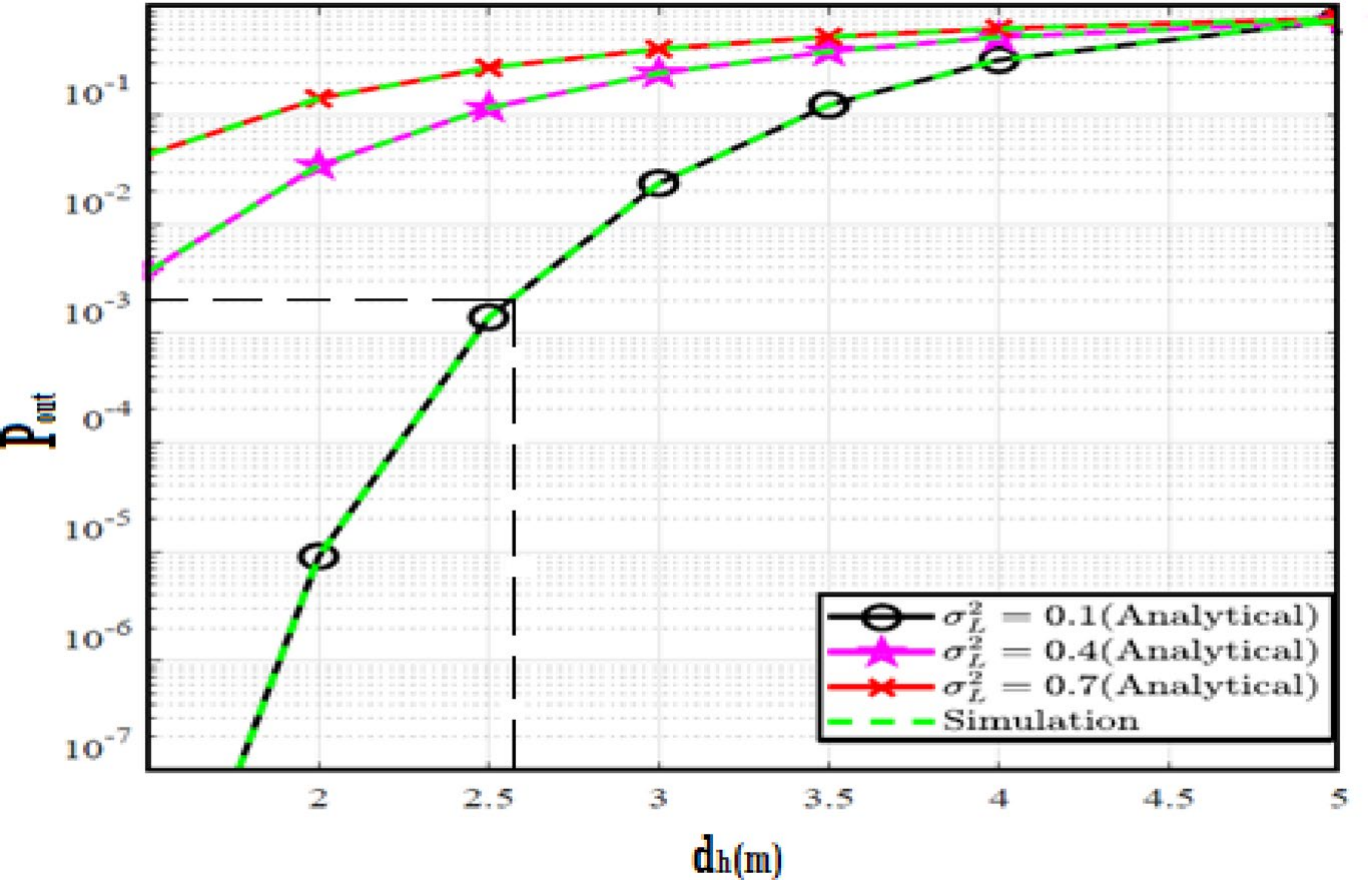
Outage Probability Versus Lateral Shift for Scenario 2 using CNN-U-Net.

**Fig. 10:**
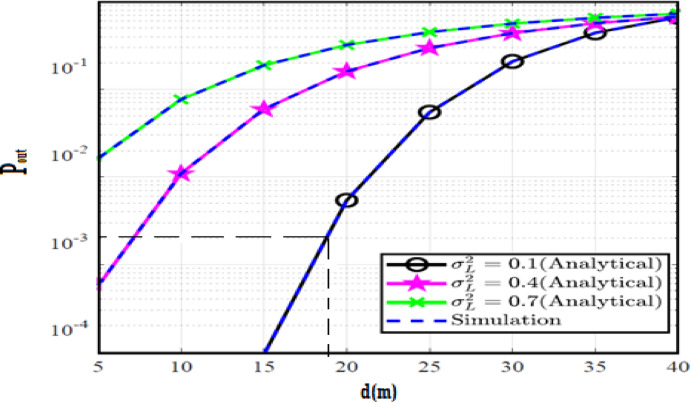
Outage Probability Versus Longitudinal Separation for Scenario 1 using CNN-U-Net.

**Fig. 11:**
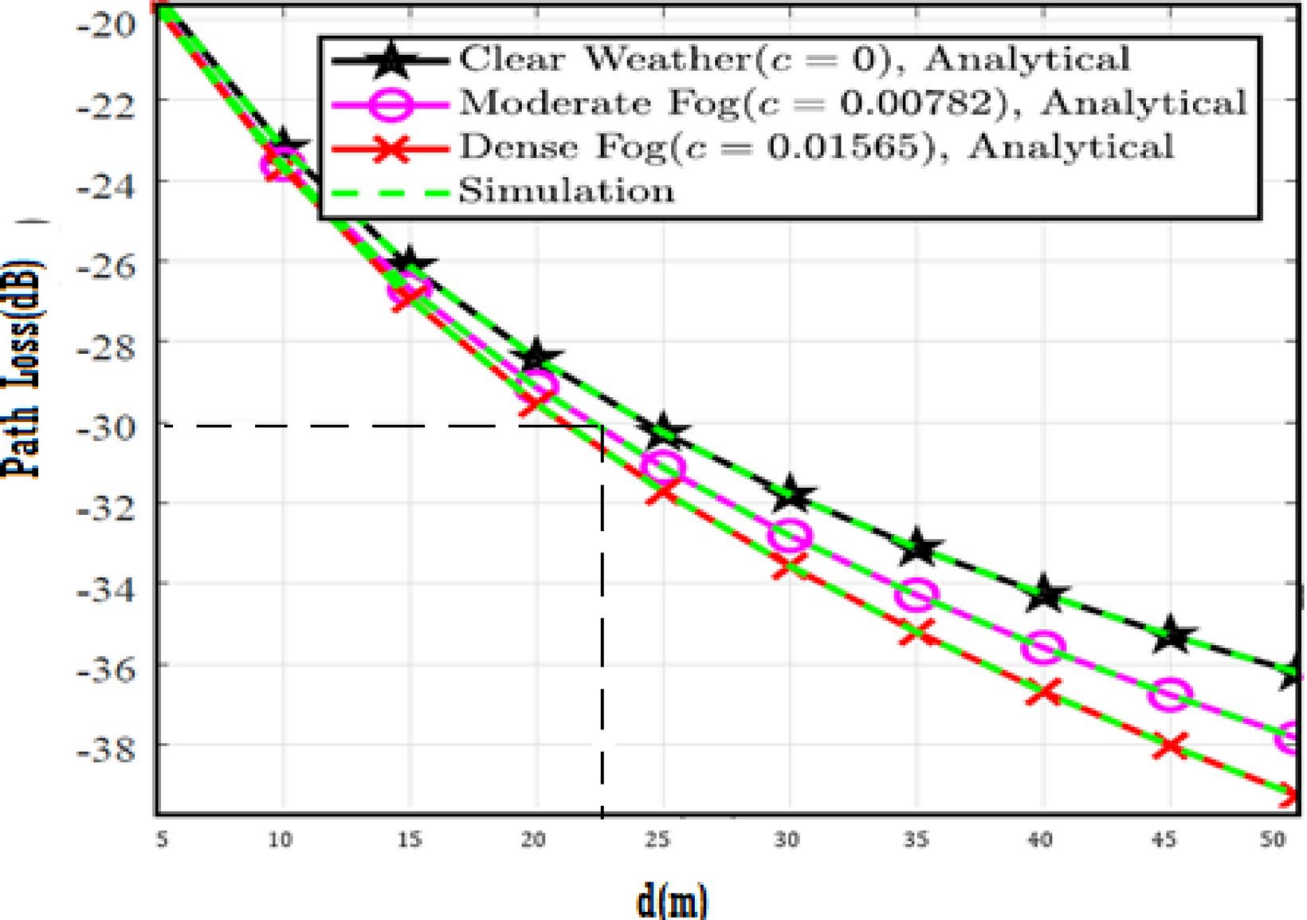
Path Loss Versus Lateral Shift for Scenario 2 using CNN-U-Net.

**Fig. 12:**
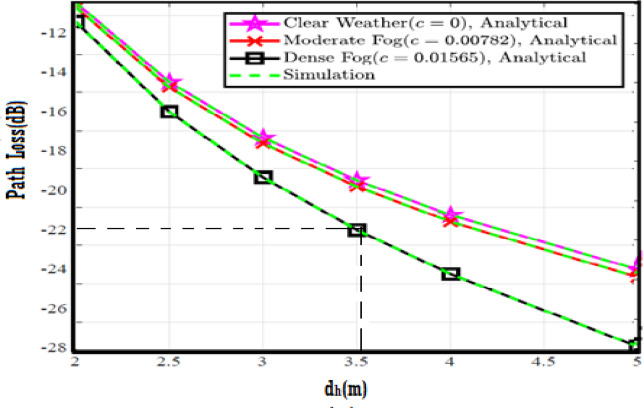
Path Loss Versus Longitudinal Separation for Scenario 1 using CNN-U-Net.

**Fig. 13:**
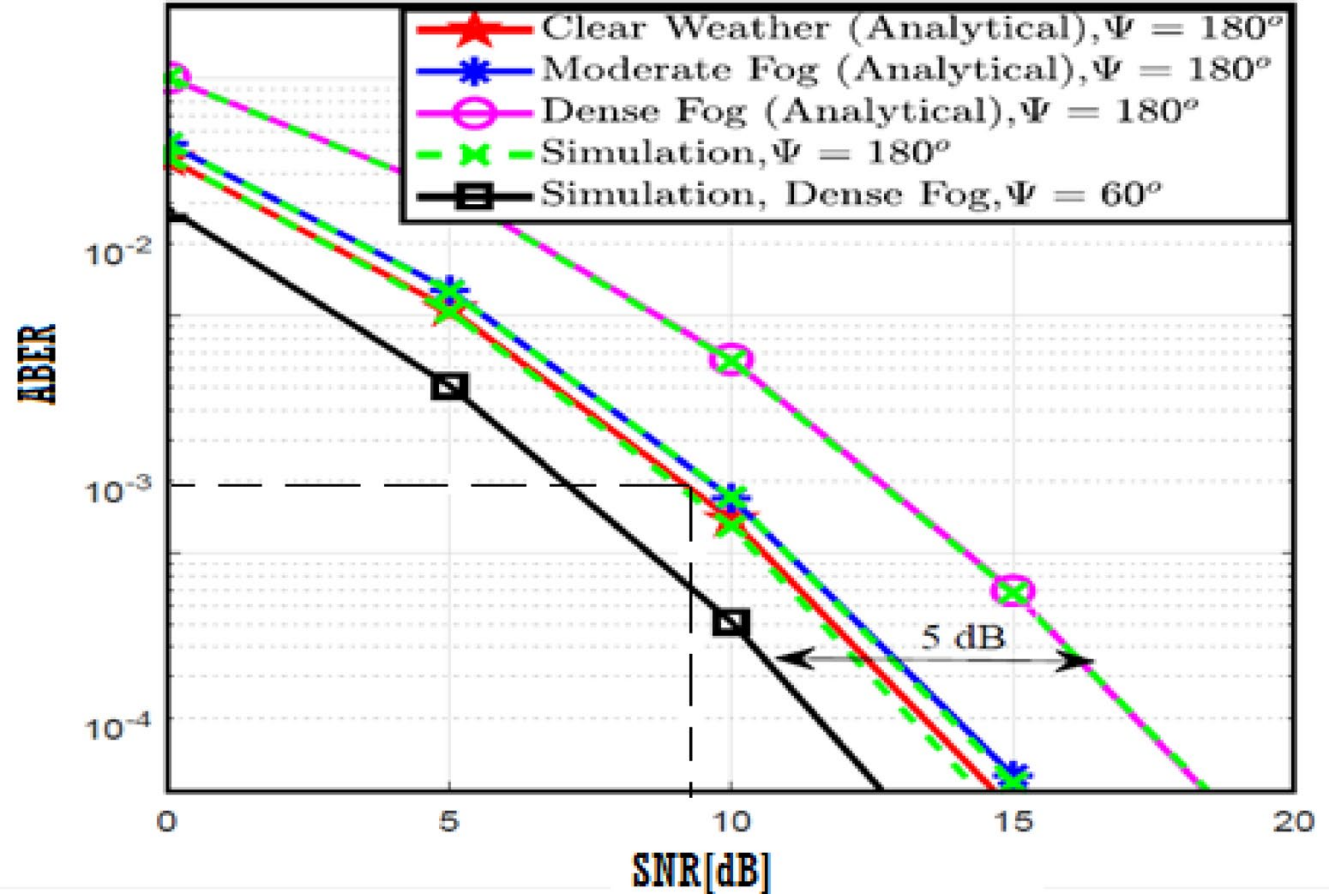
ABER Performance Versus SNR for Scenario 2 using CNN-U-Net.

**Fig. 14:**
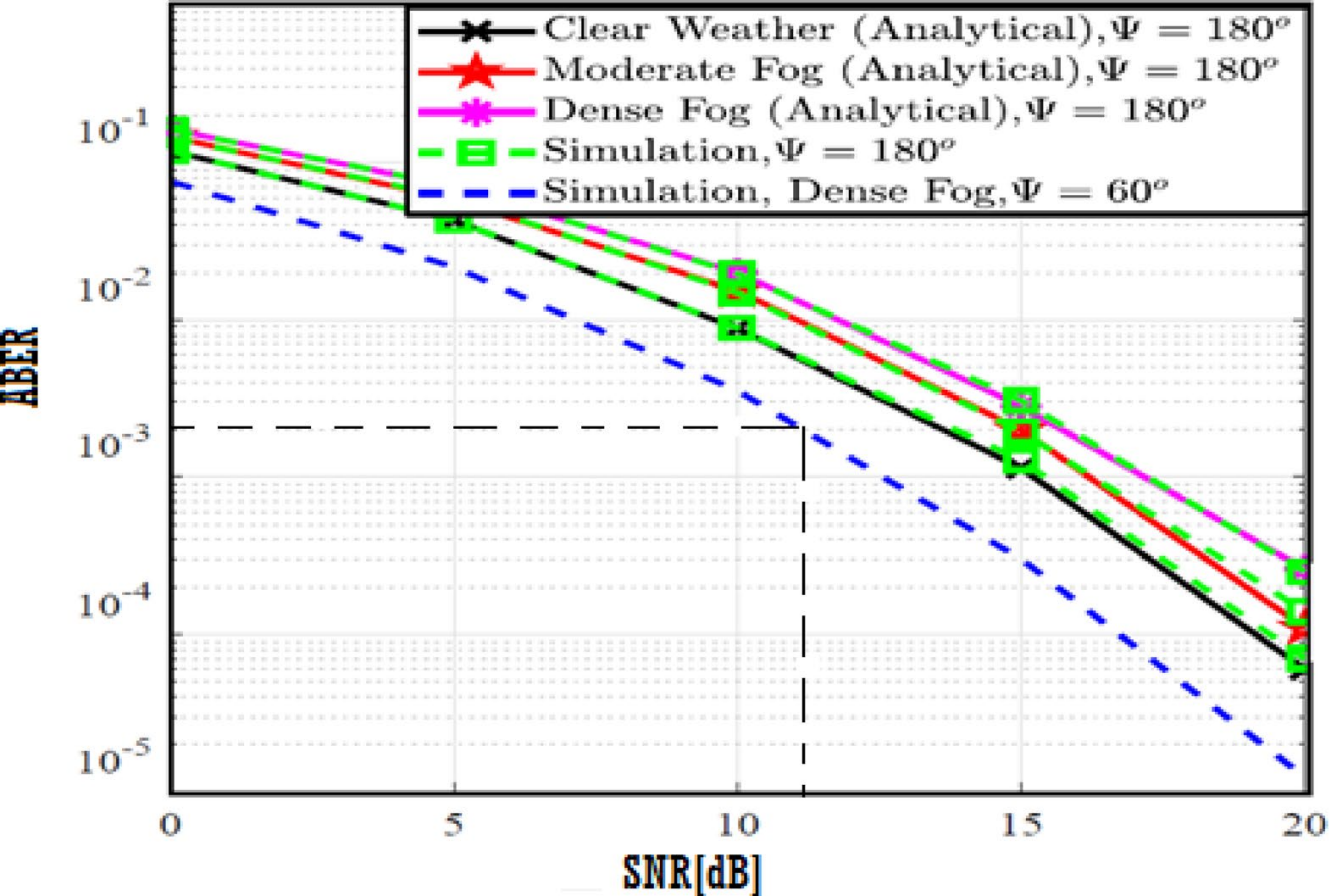
ABER Performance Versus SNR for Scenario 1 using CNN-U-Net.

The system performance is improved based on CNN-U-Net model as seen in Fig. 9. It is observed that the relation between Lateral shift of the vehicle and output power where high value of $\:{\sigma\:}_{L}^{2}$ achieves higher P_out_ for scenario 2. While the Fig. 10, illustrates the relation between Longitudinal separation between the two vehicles with output power for scenario 1 and like the pervious results, the increasing of $\:{\sigma\:}_{L}^{2}$ increase the output power. The relation between pathloss and the Lateral shift of the vehicle is opposite relation as shown in Fig. 11. For scenario 2, while increasing the Lateral shift of the vehicle, the pathloss is decreased and enhanced the performance. Figure 12 shows scenario 1 with opposite relation between pathloss and Longitudinal separation between the two vehicles. The performance of the system can be evaluated by using the relation between ABER and SNR as illustrated in Fig. 13 for scenario 2 while scenario 1 is shown in Fig. 14, for different conditions of the weather. By comparison the results by that in Ref.^18^, the improvement percentage of utilizing CNN-U-Net model is deduced in Figs. 6, 7, 8, 9, 10, 11 by (7.1%, 3.4%, 5%, 20%, 15%, 8%).

2. CNN-U-Net-GAN model

**Fig. 15:**
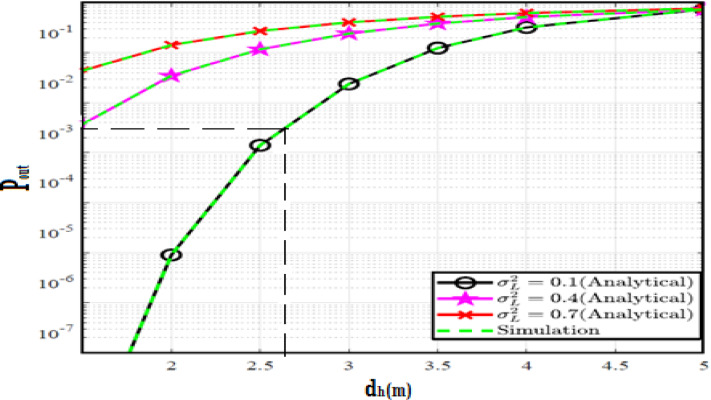
Outage Probability Versus Lateral Shift for Scenario 2 using CNN-U-Net-GAN.

**Fig. 16:**
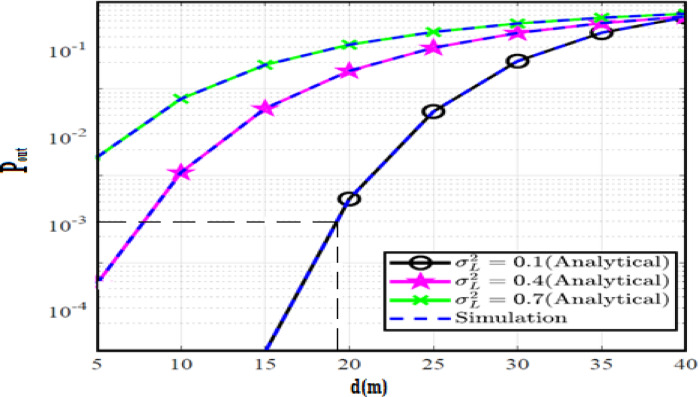
Outage Probability Versus Longitudinal Separation for Scenario 1 using CNN-U-Net-GAN.

**Fig. 17:**
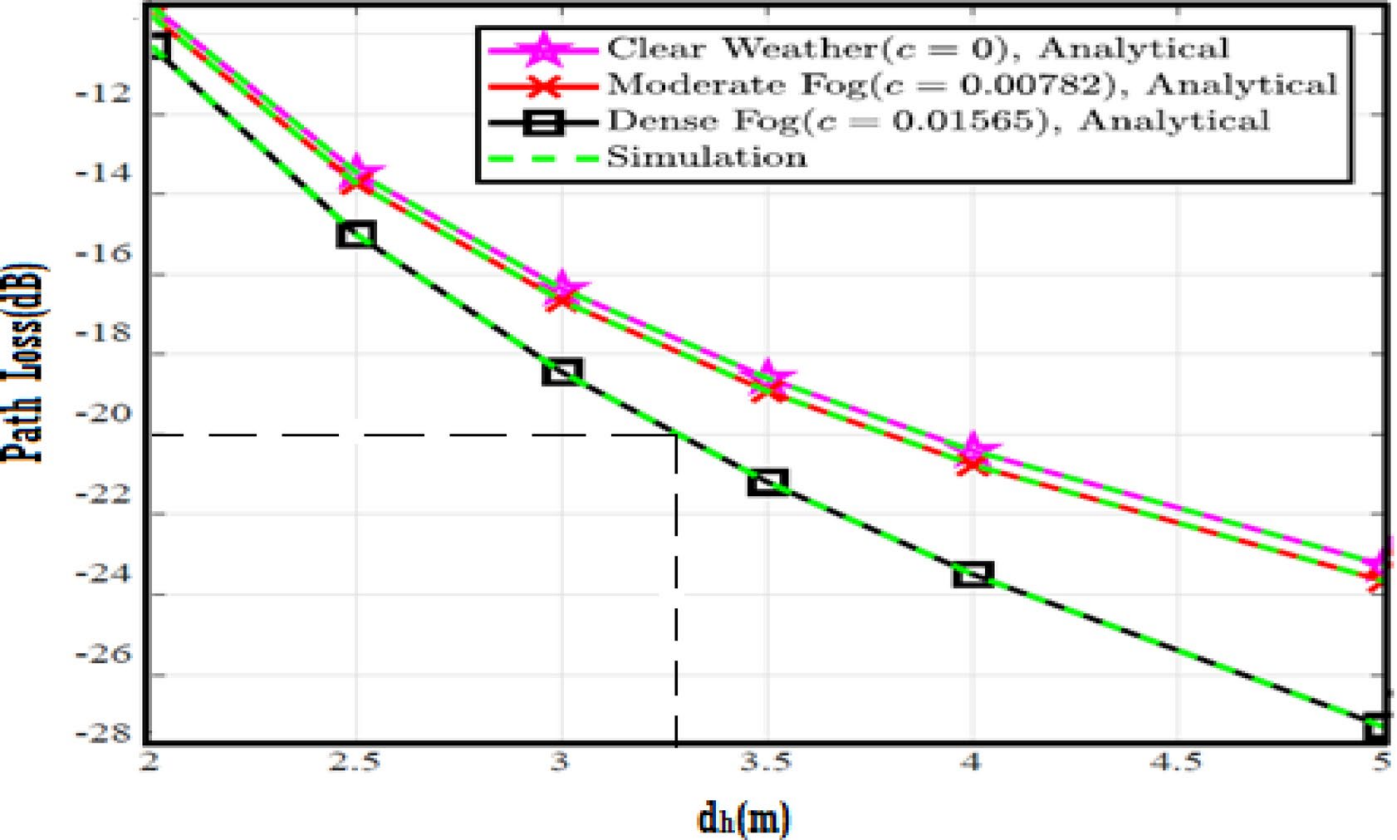
Path Loss Versus Lateral Shift for Scenario 2 using CNN-U-Net-GAN.

**Fig. 18:**
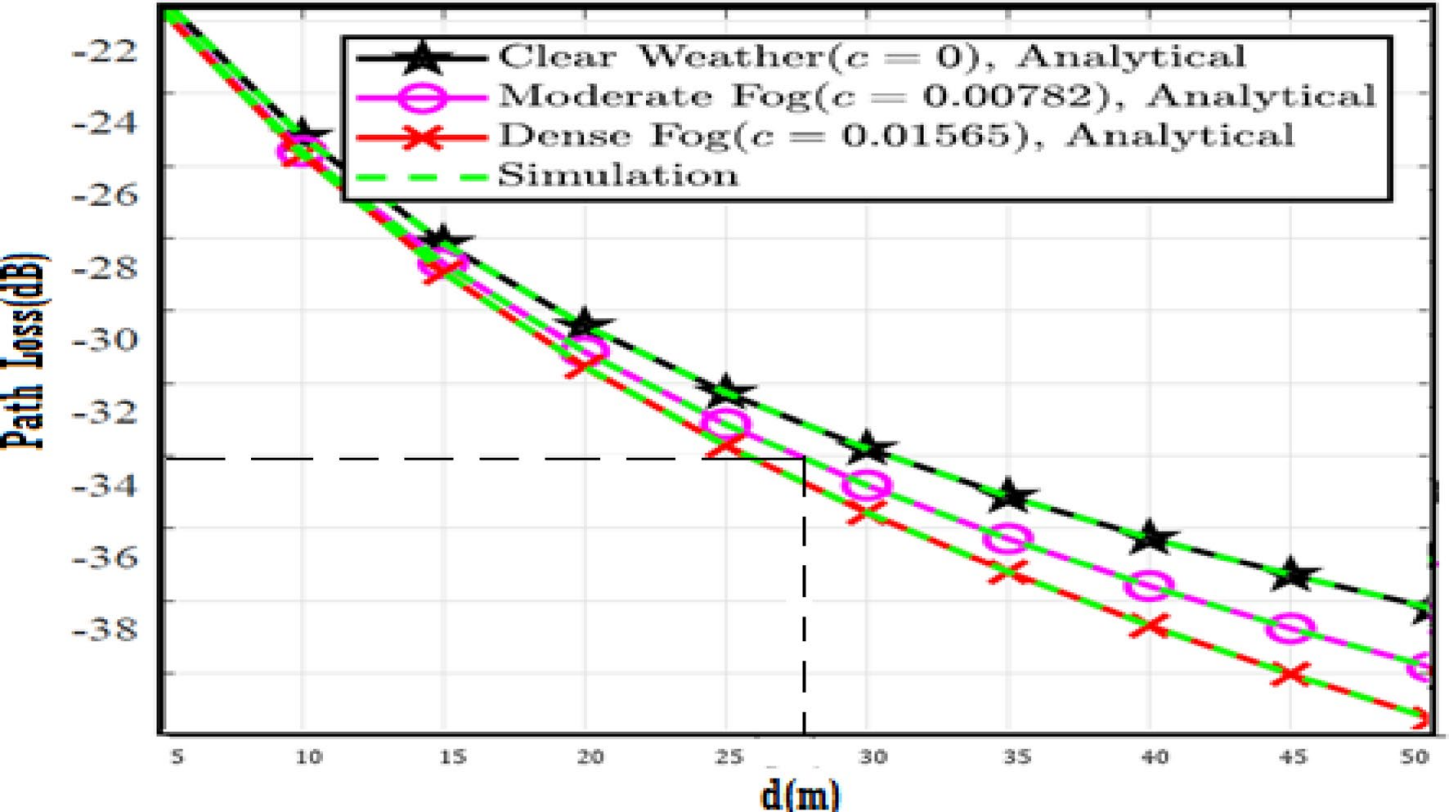
Path Loss Versus Longitudinal Separation for Scenario 1 using CNN-U-Net-GAN.

**Fig. 19:**
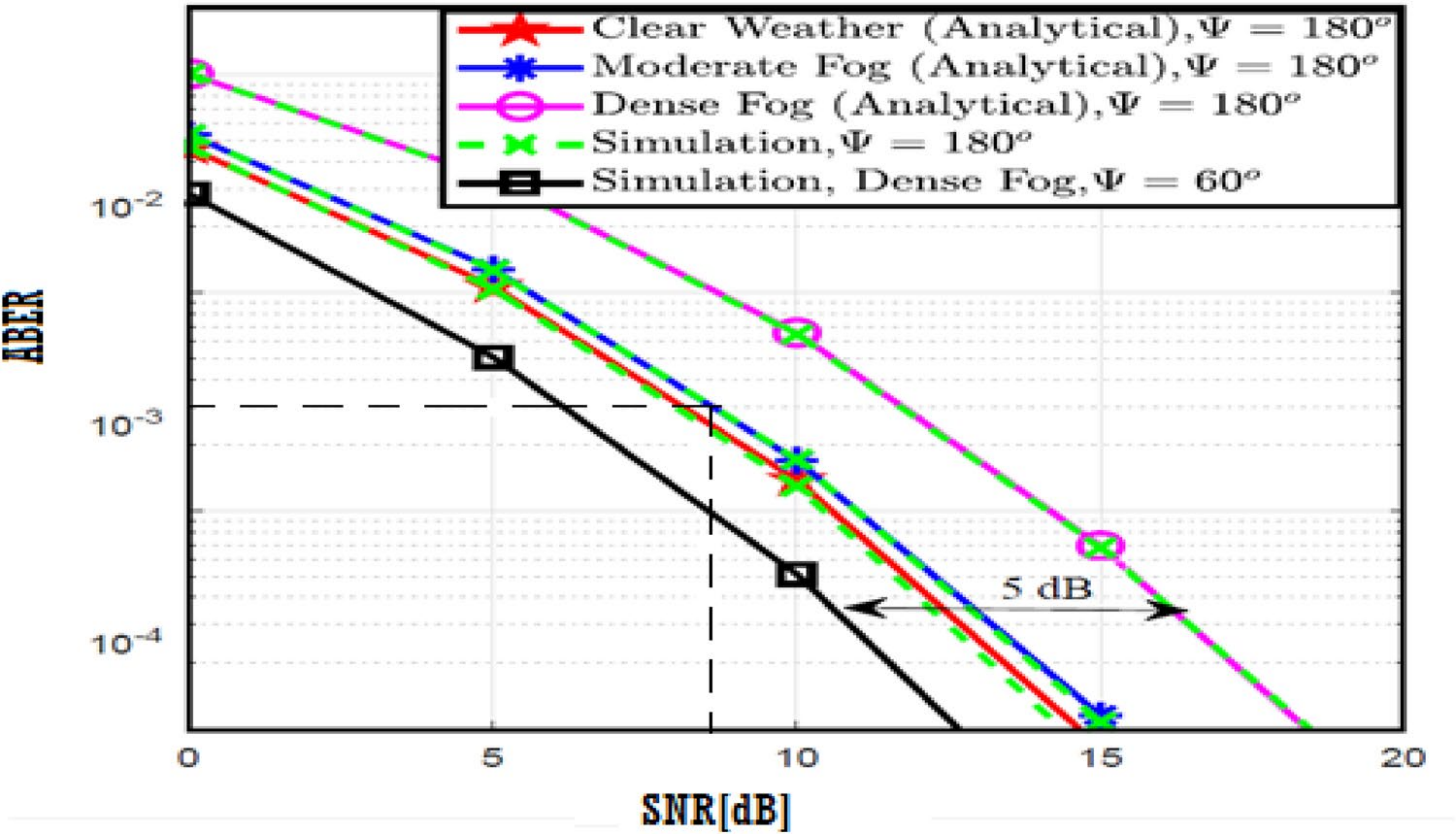
ABER Performance Versus SNR for Scenario 2 using CNN-U-Net-GAN.

**Fig. 20:**
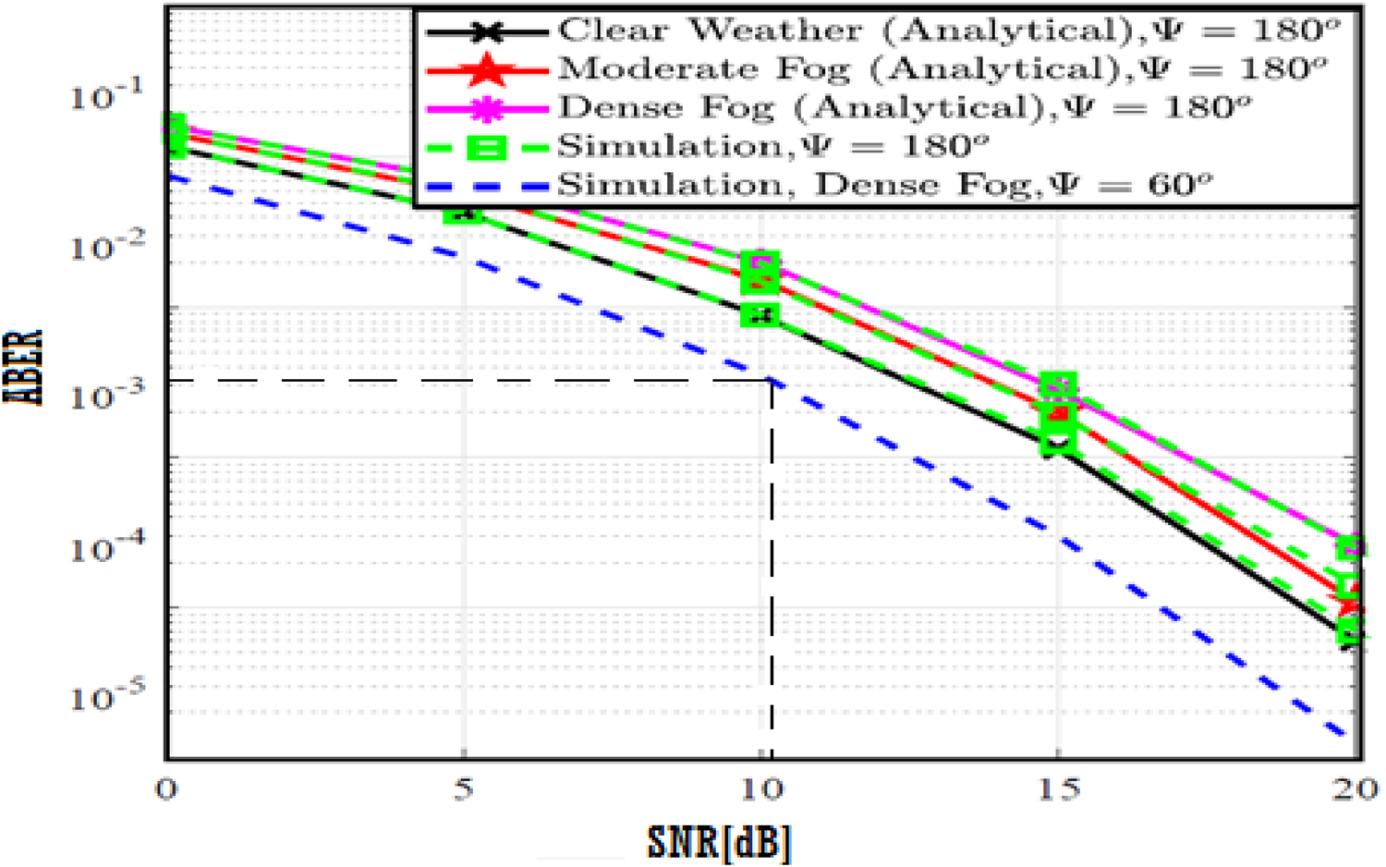
ABER Performance Versus SNR for Scenario 1 using CNN-U-Net-GAN.

Similar to the previous model, the results of the output power are shown in Figs. 15 and 16 for scenario 2 and scenario 1 respectively. The path loss relations for both scenarios are shown in Figs. 17 and 18 and the relation between ABER with SNR is shown in Figs. 19 and 20. The addition of using GAN technique increase the performance of model CNN-U-Net-GAN over CNN-U-Net by average improvement percentage by 10%.

By comparison the results by that in Ref.^18^, the improvement percentage of applying CNN-U-Net-GAN model, Figs. 15, 16, 17, 18, 19, 20, is discovered to be (8%, 14.28%, 4.28%, 30%, 25%, 25%).

3. Model CNN-U-Net-GAN-GRU.

This model outperforms both CNN-U-Net model by (12%, 24%) and CNN-U-Net-GAN model (2%, 7%) models in pathloss for two scenarios and performance of ABER respectively. While the performance of the CNN-U-Net-GRU like similar performance to the CNN-U-Net-GAN model regarding the lateral shift of the vehicle and Longitudinal separation between the two vehicles for two scenarios.

**Fig. 21:**
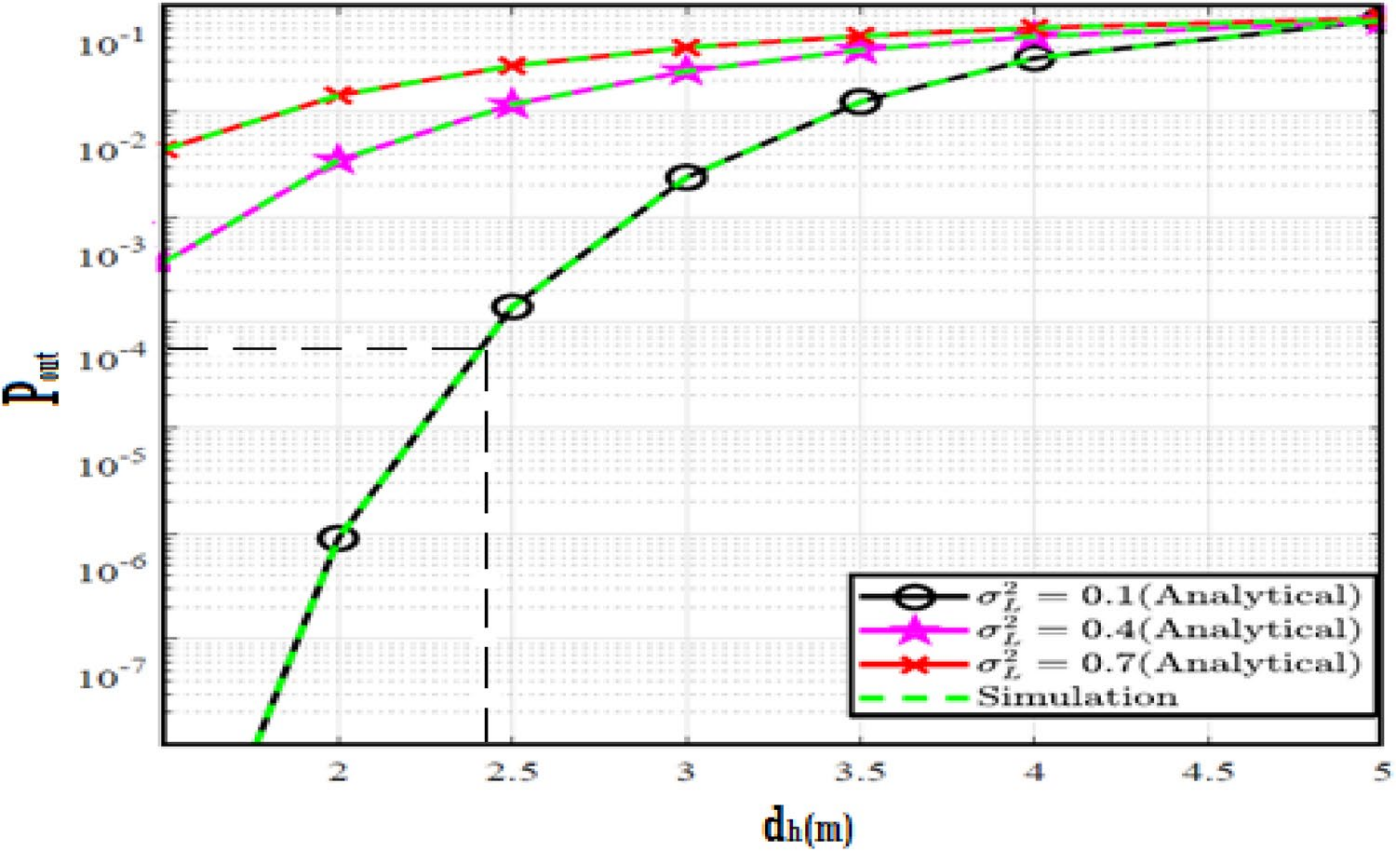
Outage Probability Versus Lateral Shift for Scenario 2 using CNN-U-Net-GAN-GRU.

**Fig. 22:**
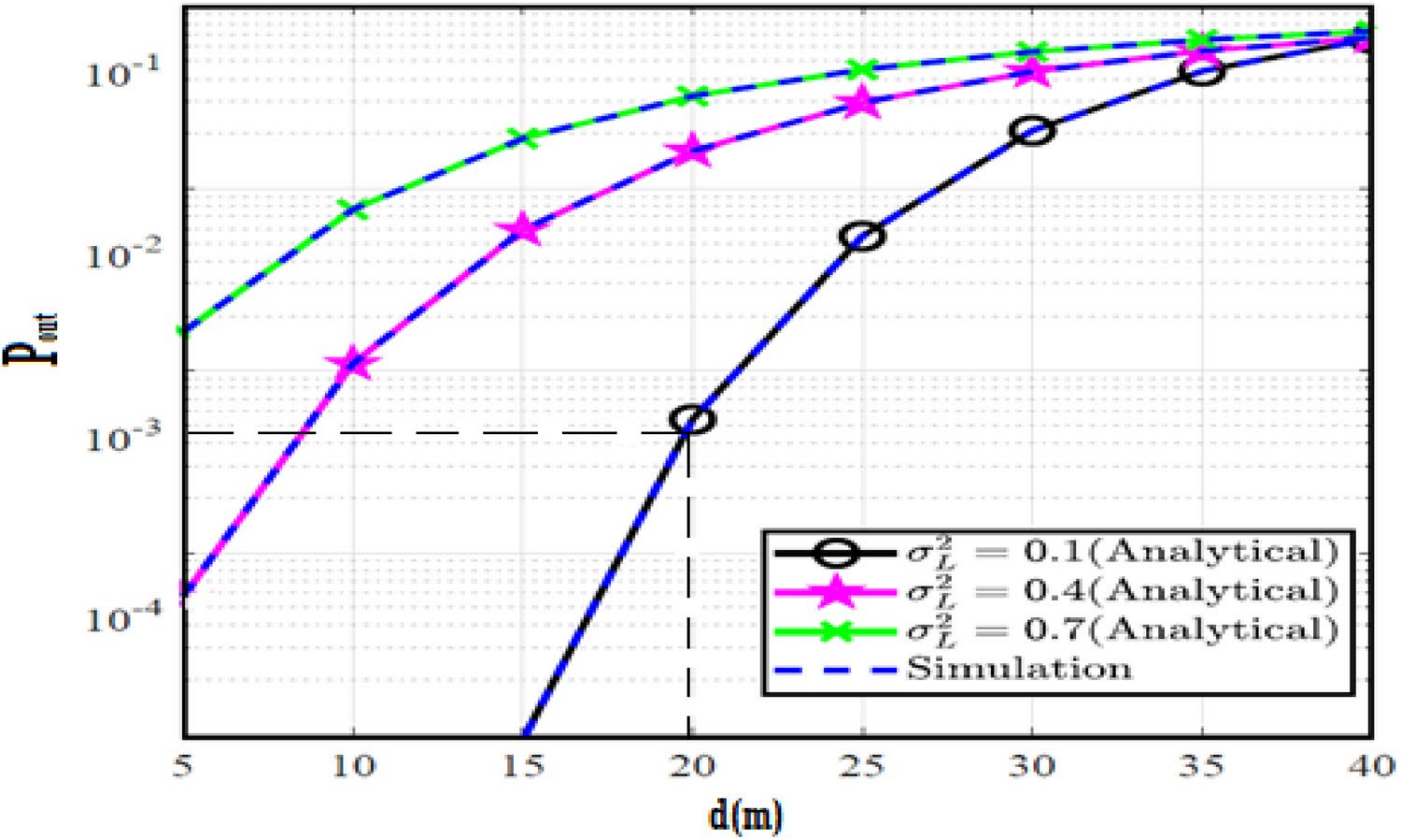
Outage Probability Versus Longitudinal Separation for Scenario 1 using CNN-U-Net-GAN-GRU.

**Fig. 23:**
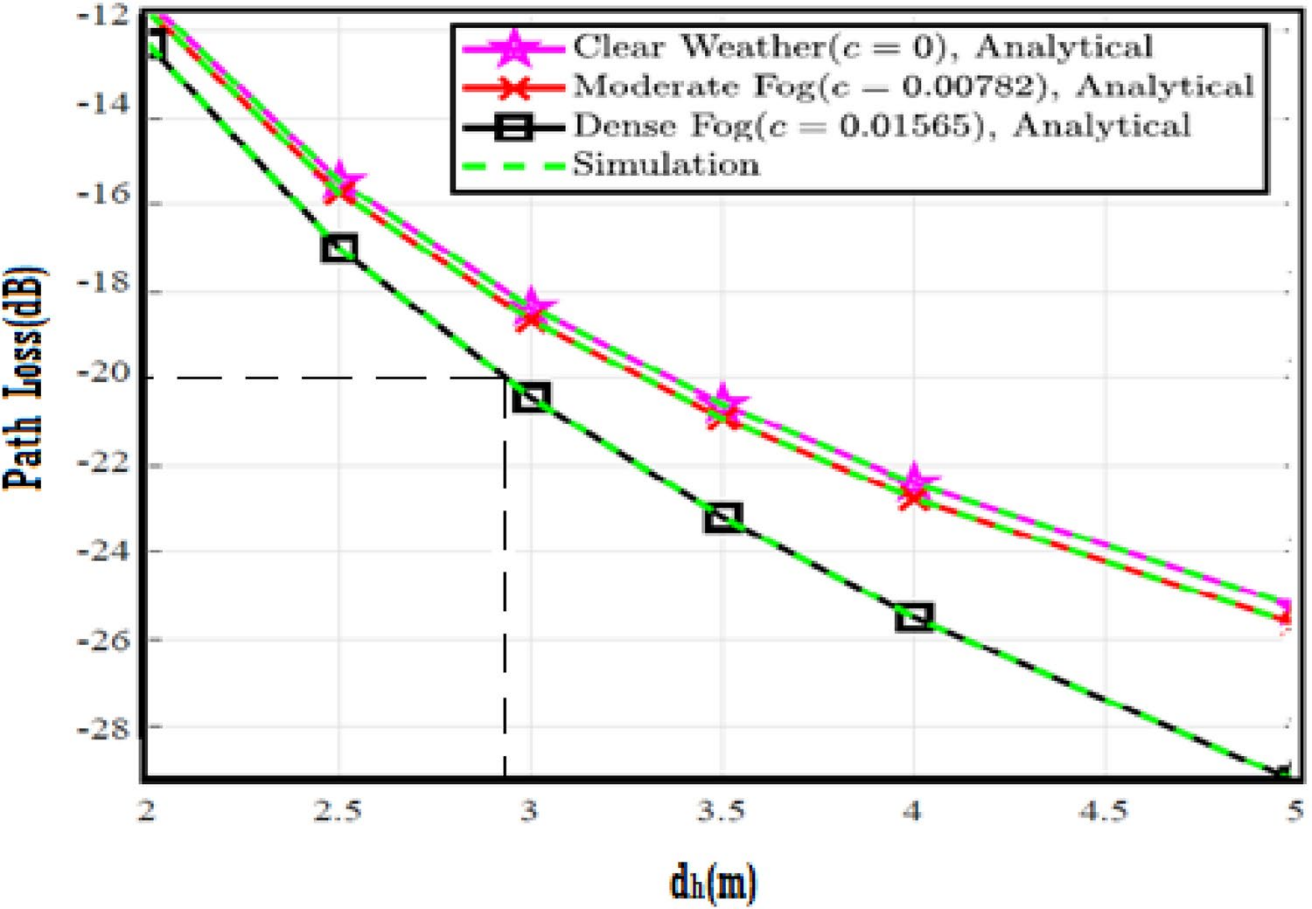
Path Loss Versus Lateral Shift for Scenario 2 using CNN-U-Net-GAN-GRU.

**Fig. 24:**
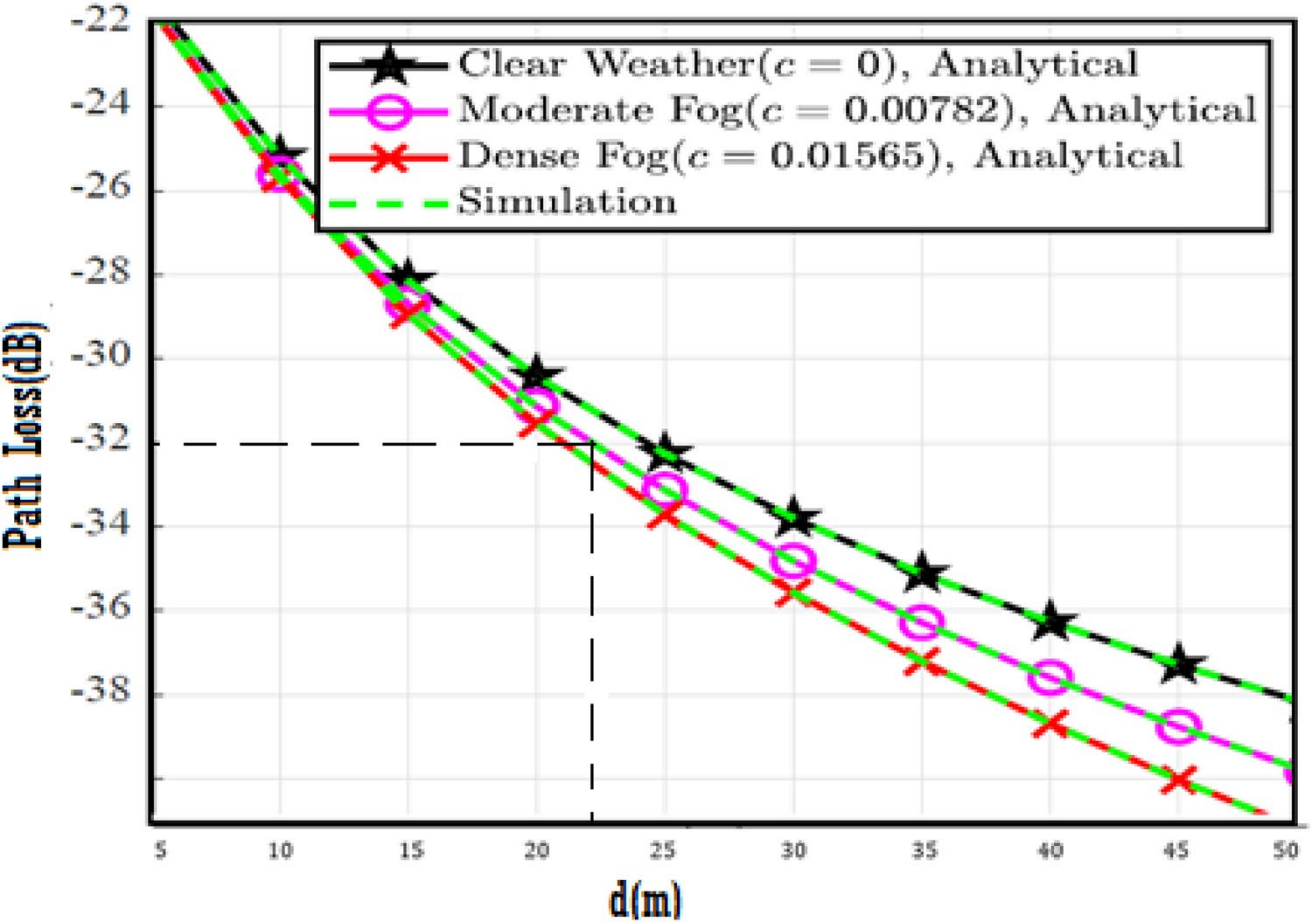
Path Loss Versus Longitudinal Separation for Scenario 1 using CNN-U-Net-GAN-GRU.

**Fig. 25:**
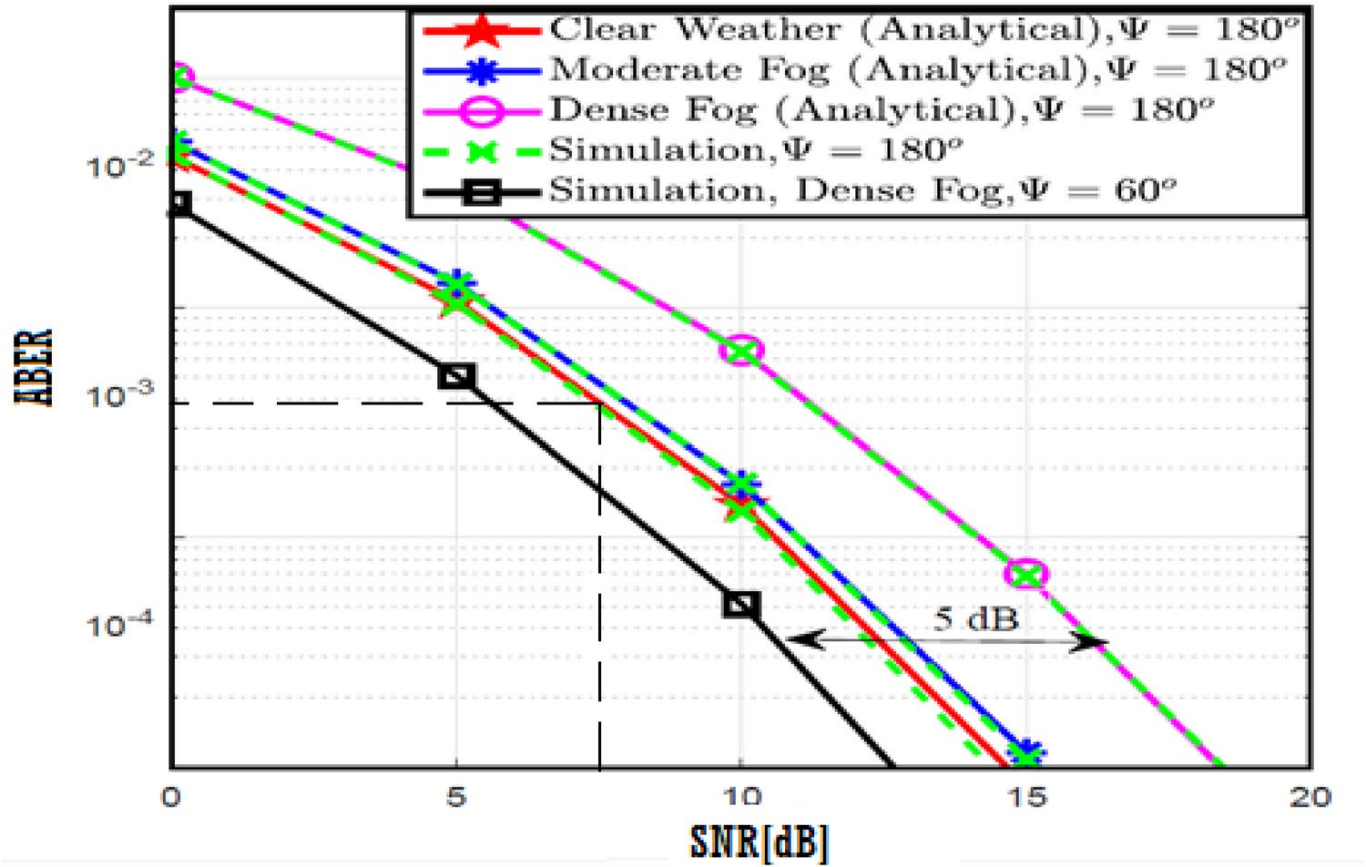
ABER Performance Versus SNR for Scenario 2 using CNN-U-Net-GAN-GRU.

**Fig. 26:**
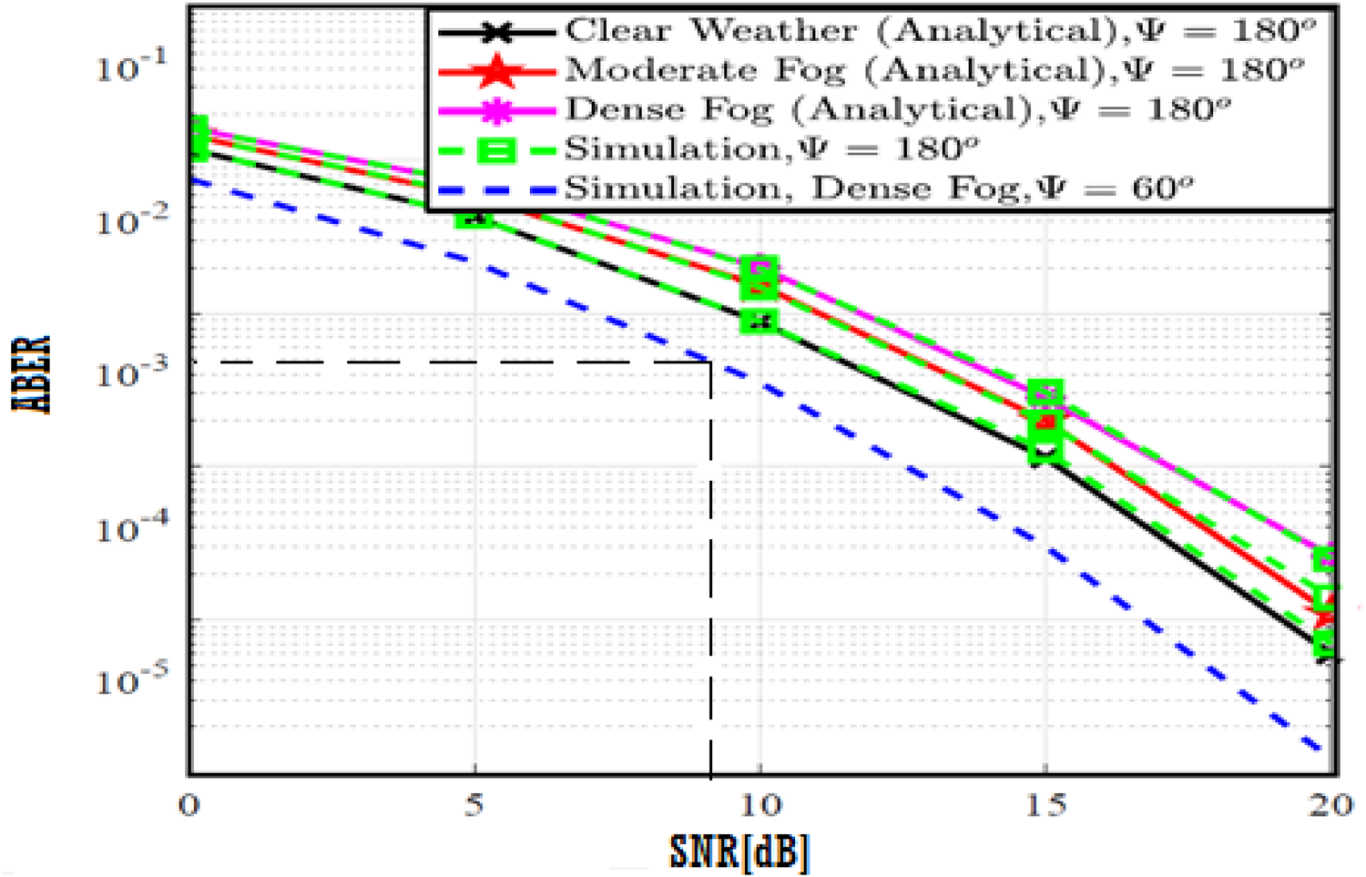
ABER Performance Versus SNR for Scenario 1 using CNN-U-Net-GAN-GRU.

By comparison the results with Ref.^18^, the CNN-U-Net-GRU performance is evaluated in Figs. 21, 22, 23, 24, 25, 26 by (8%, 14.28%, 13.84%, 32%, 37.5%, 32%).

4. CNN-U-Net-GAN-GRU-DDAE

The CNN-U-Net-GAN-GRU-DDAE model is hybrid model for the three previous models where it outperforms the other models and achieves the highest results for all of the metrics; pathloss, ABER and Lateral shift of the vehicle and Longitudinal separation between the two vehicles for two scenarios.

**Fig. 27:**
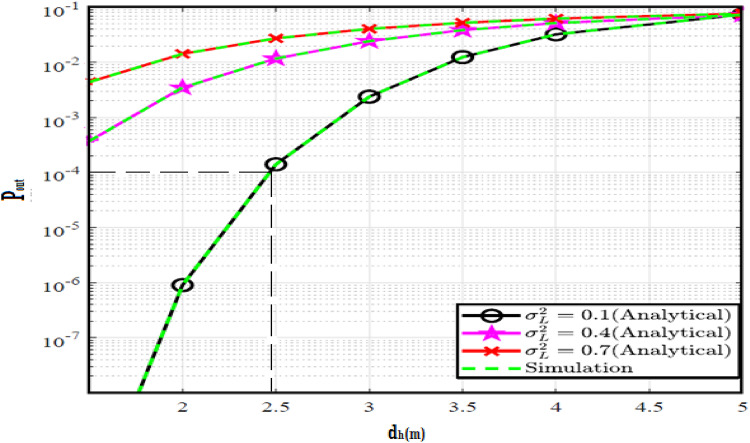
Outage Probability Versus Lateral Shift for Scenario 2 using CNN-U-Net-GAN-GRU-DDAE.

**Fig. 28:**
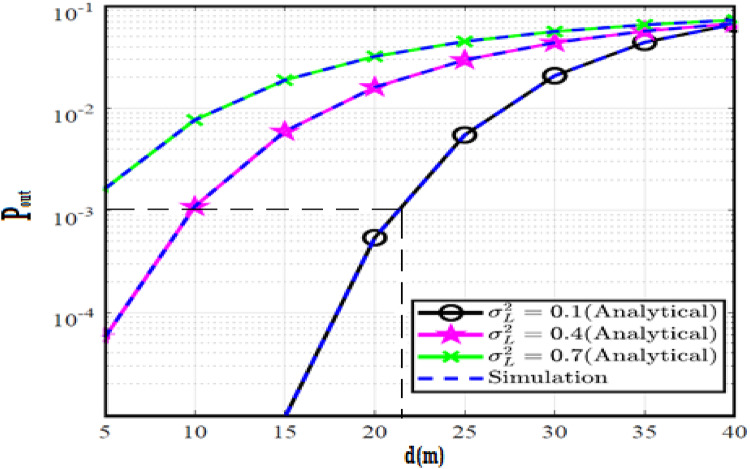
Outage Probability Versus Longitudinal Separation for Scenario 1 using CNN-U-Net-GAN-GRU-DDAE.

**Fig. 29:**
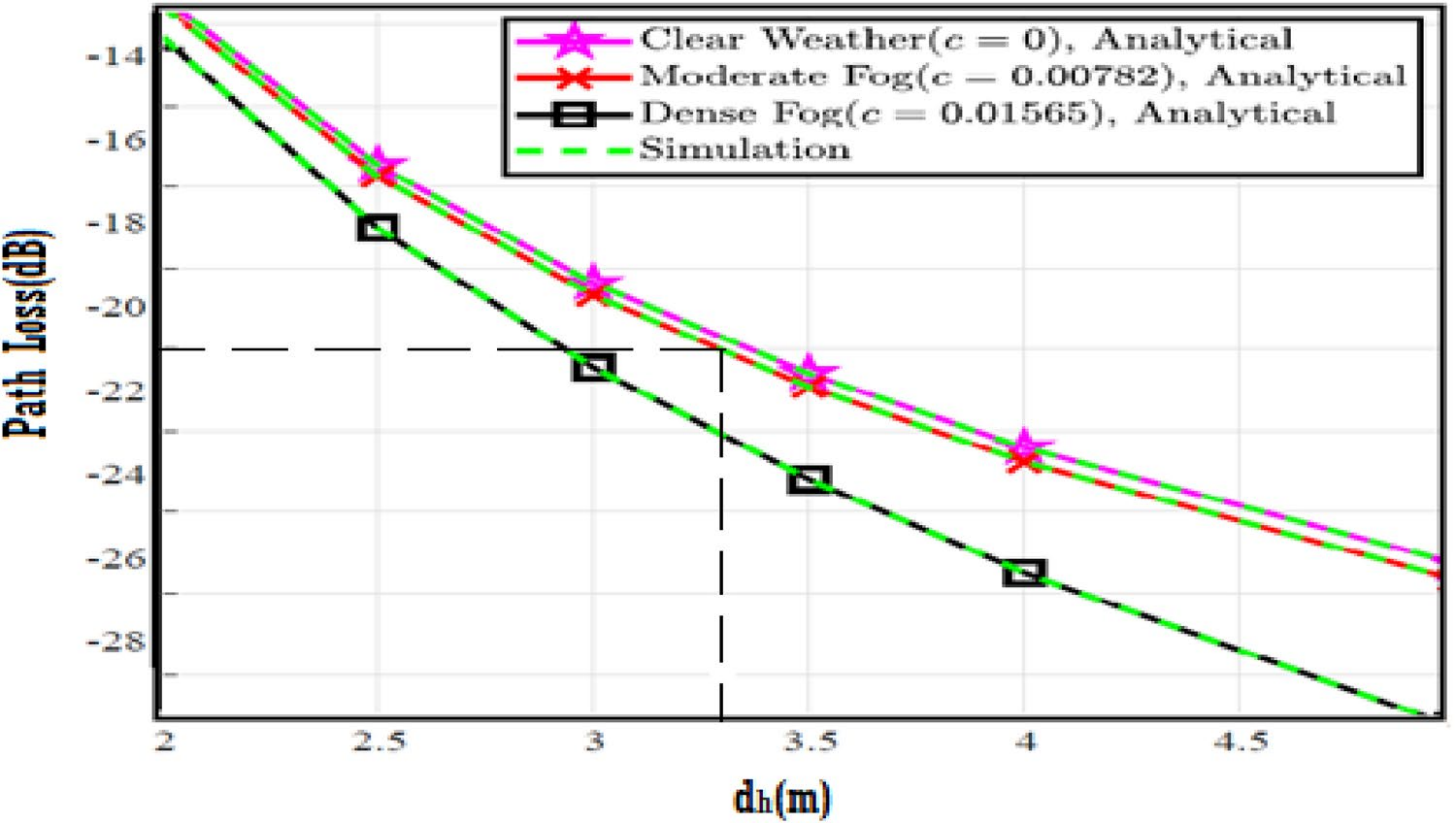
Path Loss Versus Lateral Shift for Scenario 2 using CNN-U-Net-GAN-GRU-DDAE.

**Fig. 30:**
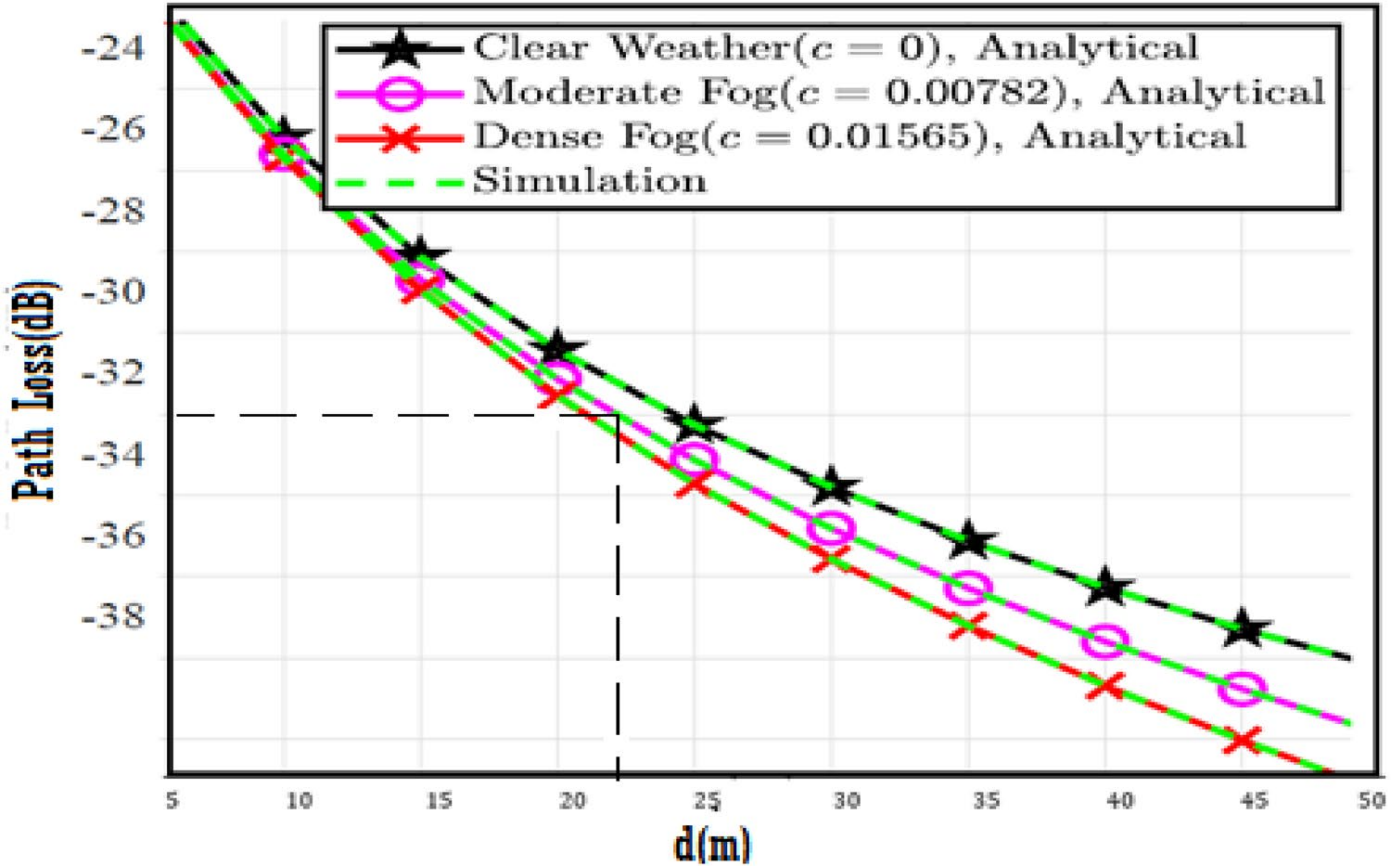
Path Loss Versus Longitudinal Separation for Scenario 1 using CNN-U-Net-GAN-GRU-DDAE.

**Fig. 31:**
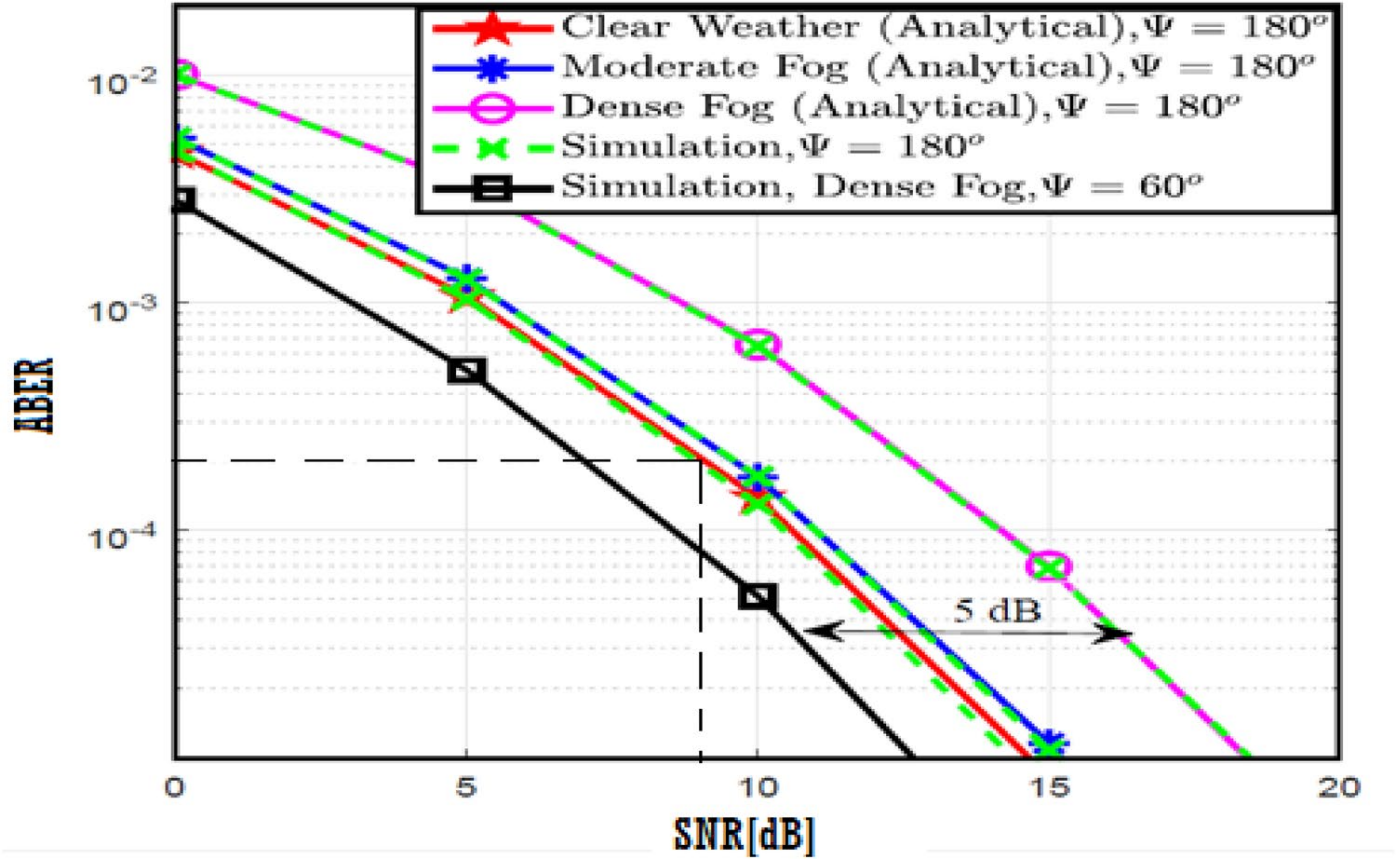
ABER Performance Versus SNR for Scenario 2 using CNN-U-Net-GAN-GRU-DDAE.

**Fig. 32:**
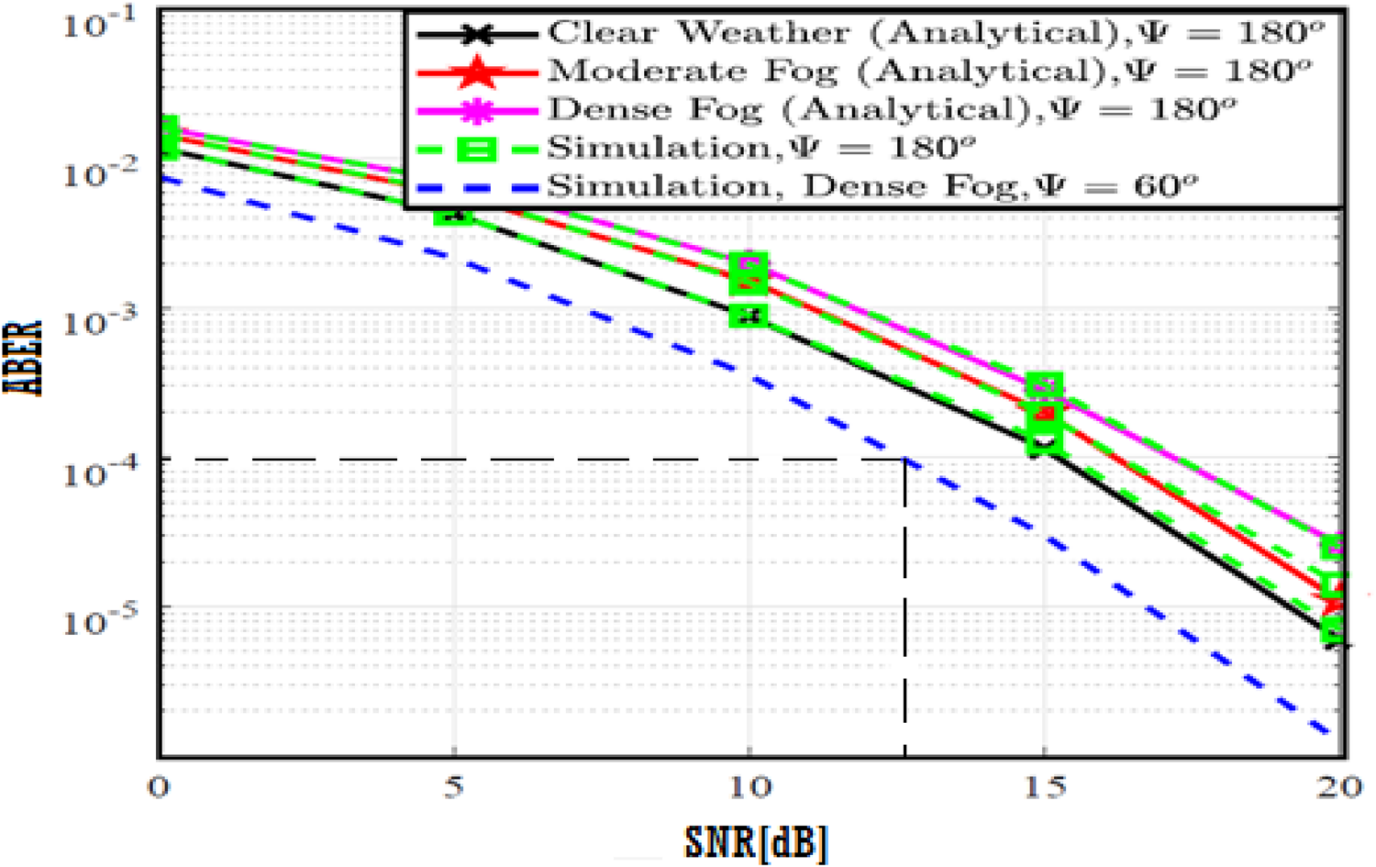
ABER Performance Versus SNR for Scenario 1 using CNN-U-Net-GAN-GRU-DDAE.

By comparison the results with Ref.^18^, the CNN-U-Net-GRU-DDAE performance for Figs. 27, 28, 29, 30, 31, 32 is calculated by (12, 20%, 18.6%, 37.5%, 62.5%, 40%).

Table 7 shows the summarization of the comparison between this work and Ref.^18^ for Figs. 9,10,11,12,13,14,15,16,17,18,19,20,21,22,23,24,25,26,27,28,29,30,31,32.

**Table 7:** Comparison between models and results of^18^.

	Lateral shift of the vehicle	Longitudinal separation	Pathloss Scenario 2	Pathloss Scenario 1	ABER Scenario 2	ABER Scenario 1
CNN-U -Net	7.1%	3.4%	5%	20%	15%	8%
CNN-U -Net-GAN	8%	14.28%	4.28%	30%	25%	25%
CNN-U -Net-GAN-GRU	8%	14.28%	13.84%	32%	37.5%	32%
CNN-U -Net-GAN-GRU-DDAE	12%	20%	18.6%	37.5%	62.5%	40%

## Conclusion

This work interest in enhancing the V2V–VLC model performance for optical wireless communication systems. Based on the results in Ref.^18^, it is observed that the proposed framework achieves best results for different metrics. Deep learning techniques are used to achieve the target and applied four models; CNN-U -Net, CNN-U -Net-GAN, CNN-U -Net-GAN-GRU, CNN-U -Net-GAN-GRU-DDAE.

In the discussion section, the numerical results are discussed with respect to the previous work and compare the models with each other. The scenarios used in this work are applied as shown in system model and the metrics used to evaluate the performance are lateral shift of the vehicle, longitudinal separation, pathloss and ABER. The CNN-U -Net-GAN-GRU-DDAE is hybrid model concluding three other models to achieve the best results. As shown in Table 7, the improvement percentages is concluded for the system V2V – VLC model with model CNN-U -Net-GAN-GRU-DDAE by comparing its results with that in Ref.^18^. The improvement percentage of model CNN-U -Net-GAN-GRU-DDAE is 12% for Lateral shift of the vehicle and 20% for Longitudinal separation, also, (18.6%, 37.5%) for pathloss metric in scenario 2 and scenario 1 respectively. While the improvement percentages for ABER metric for scenario 2 and scenario 1 respectively are 62.5% and 40%. On another side, it is noticed that CNN-U -Net-GAN-GRU-DDAE model outperforms other models CNN-U -Net, CNN-U -Net-GAN, CNN-U -Net-GAN-GRU.

It is acknowledged that the performance of the suggested CNN-U-Net-GAN-GRU-DDAE architecture for interference cancellation and BER reduction in V2V communication systems is evaluated in this study exclusively using simulation-based results. Simulations provide a controlled and reproducible setting for testing models under various channel conditions, which makes them a useful initial step for comparative analysis and proof-of-concept.

The results contain the weather conditions and two scenarios contains the different cases of mobility of vehicle but the limitations in this study that doesn’t include the worst weather conditions to deduce the system performance in this case, also, the combining models, CNN-U -Net, CNN-U -Net-GAN, CNN-U -Net-GAN-GRU, CNN-U -Net-GAN-GRU-DDAE are very complicated and have complex equations, furthermore the long run time to apply the program coding in these modes is very high.

The future work for deploying in the real world can be determined by specific points as the following:

- Suppose different environments with different weather conditions.
- Investigate other models to enhance the system performance of V2V- VLC model.
- Attempt to decrease the complexity of models by investigating different techniques and thus will decrease the long run time of code programming.
- Additional scenarios for parked vehicles will be considered.

## Data Availability

The datasets used and/or analysed during the current study available from the corresponding author on reasonable request.
